# Revisiting Solid–Solid Phase Transitions in Sodium and Potassium Tetrafluoroborate for Thermal Energy Storage

**DOI:** 10.1021/acs.chemmater.3c02039

**Published:** 2024-01-31

**Authors:** Sumit Konar, Gertruda Zieniute, Elliot Lascelles, Beth Wild, Andreas Hermann, Yi Wang, Robert J. Quinn, Jan-Willem G. Bos, Andrew Fitch

**Affiliations:** † Joseph Banks Laboratories, School of Chemistry, University of Lincoln, Lincoln LN6 7DL, United Kingdom; ‡ Centre for Science at Extreme Conditions and SUPA, School of Physics and Astronomy, The University of Edinburgh, Edinburgh EH9 3FD, United Kingdom; § School of Chemical Engineering, University of Birmingham, Birmingham B15 2TT, United Kingdom; ∥ Institute of Chemical Sciences, School of Engineering & Physical Sciences, Heriot-Watt University, Edinburgh EH14 4AS, United Kingdom; ⊥ EaStCHEM School of Chemistry, University of St Andrews, North Haugh, St Andrews KY16 9ST, United Kingdom; # European Synchrotron Radiation Facility, 71 avenue des Martyrs, Grenoble 38000, France

## Abstract

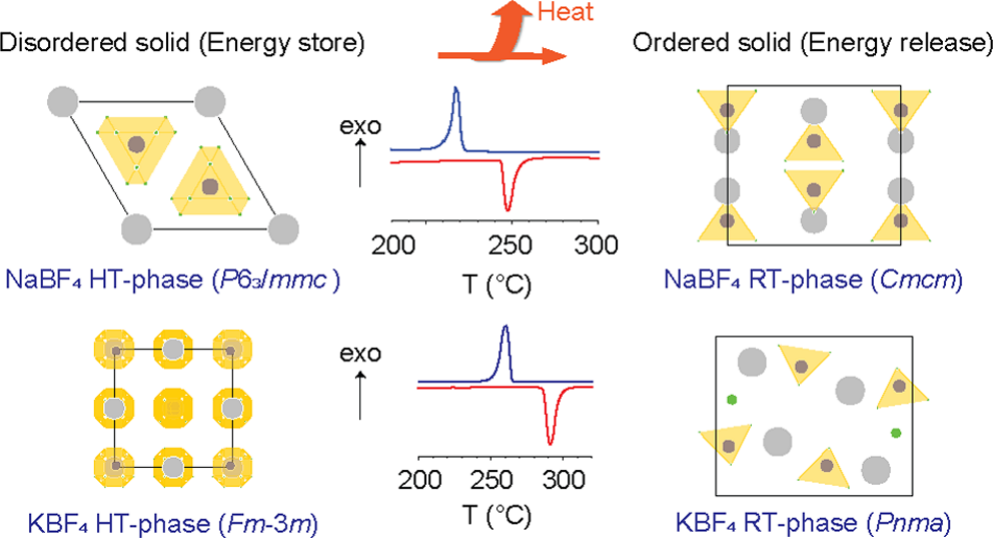

In situ synchrotron powder X-ray diffraction (PXRD) study was conducted on sodium and potassium tetrafluoroborate (NaBF_4_ and KBF_4_) to elucidate structural changes across solid–solid phase transitions over multiple heating–cooling cycles. The phase transition temperatures from diffraction measurements are consistent with the differential scanning calorimetry data (∼240 °C for NaBF_4_ and ∼290 °C for KBF_4_). The crystal structure of the high-temperature (HT) NaBF_4_ phase was determined from synchrotron PXRD data. The HT disordered phase of NaBF_4_ crystallizes in the hexagonal, space group *P*6_3_/*mmc* (no. 194) with *a* = 4.98936(2) Å, *c* = 7.73464(4) Å, *V* = 166.748(2) Å^3^, and *Z* = 2 at 250 °C. Density functional theory molecular dynamics (MD) calculations imply that the *P*6_3_/*mmc* is indeed a stable structure for rotational NaBF_4_. MD simulations reproduce the experimental phase sequence upon heating and indicate that F atoms are markedly more mobile than K and B atoms in the disordered state. Thermal expansion coefficients for both phases were determined from high-precision lattice parameters at elevated temperatures, as obtained from Rietveld refinement of the PXRD data. Interestingly, for the HT-phase of NaBF_4_, the structure (upon heating) contracts slightly in the *a*–*b* plane but expands in the *c* direction such that overall thermal expansion is positive. Thermal conductivities at room temperature were measured, and the values are 0.8–1.0 W m^–1^ K^–1^ for NaBF_4_ and 0.55–0.65 W m^–1^ K^–1^ for KBF_4_. The thermal conductivity and diffusivity showed a gradual decrease up to the transition temperature and then rose slightly. Both materials show good thermal and structural stabilities over multiple heating/cooling cycles.

## 1 Introduction

Almost half of the final energy consumed in the world is used to provide heating/cooling. The intermittent nature of renewable energy requires the development of cost-efficient heat storage materials. There are essentially three methods for thermal energy storage: chemical, latent, and sensible.^1^ Despite chemical heat storage showing the highest potential due to high energy densities, currently there are substantial safety concerns and engineering challenges because of their complexity, uncertainty, and lack of a suitable material for chemical storage. While chemical heat storage technology is still at the laboratory stage, sensible and latent heat technologies are mature and already commercialized.^2^ Latent heat storage or so-called phase-change materials (PCMs) have been receiving considerable attention over sensible storage for various thermal energy storage applications.^3,4^ First, the energy density is typically much higher [e.g., sodium acetate trihydrate^5^ (CH_3_COONa·3H_2_O) 250 J/g at 58 °C; erythritol (HO(CH_2_)(CHOH)_2_(CH_2_)OH) 314 J/g at 118 °C; molten sodium nitrate (NaNO_3_) 175 J/g at 307 °C]. Second, the energy storage and release processes usually occur at a constant temperature, which means less wasted energy than sensible storage solely driven by a temperature gradient, and that can be advantageous for targeting a specific operating temperature. PCMs are not only limited to solid–liquid changes; a few solid–solid phase transitions are also known.

Solid–solid PCMs (ss-PCMs) present several advantages over conventional solid–liquid PCMs (e.g., salt hydrates, sugar alcohols, and molten salts) including safety (no spillage of hot liquid), lower thermal expansion, lower corrosiveness, and no need for encapsulation.^6^ Solid–solid transitions occur from room-temperature ordered phases to orientationally disordered high-temperature phases that lie at the boundary between liquids and solids, and the large latent heat is associated with the strong rotational motions of molecules. Few highly symmetric organic polyols^7^ (pentaerythritol (Δ*H* = 300 J/g at 184 °C), neopentylglycol (Δ*H* = 130 J/g at 42 °C), pentaglycerine (Δ*H* = 190 J/g at 81 °C), etc.), have been investigated. Organic ss-PCMs have lower density and relatively low thermal conductivity (0.1–0.3 W m^–1^ K^–1^) largely affecting charging/discharging rates.

Inorganic SS-PCMs with higher density and good thermal conductivities have been previously overlooked with regard to medium-/high-temperature (>200 °C) heat storage applications. This medium-/high-temperature heat storage has good potential since, for example, many industries including pulp and paper and iron and steel produce waste heat at 200–500 °C.^8^ A few inorganic sulfates are known to undergo transitions from a low-temperature ordered phase to orientationally disordered high-temperature phase(s) with large changes in enthalpy. The inorganic SS-PCM that has been most investigated is lithium sulfate (Li_2_SO_4_),^9,10^ as its crystalline transformation takes place at temperatures appropriate for CSP technologies. Na_2_SO_4_ has been reported to exist in five polymorphous forms labeled I–V. The structural transformation in sodium sulfate (Na_2_SO_4_) is still a subject of debate.^11^ The phase transition enthalpy of Na_2_SO_4_ is not large (Δ*H* = 50 J/g), but its transition temperature is quite low (240 °C); thus, it may be used at a relatively low temperature.^12^ The low-temperature orthorhombic form of K_2_SO_4_ (*Pmcn*) transforms to the high-temperature hexagonal form of K_2_SO_4_ (*P*6_3_/*mmc*) at 583 °C at Δ*H* = 25–40 J/g. High-temperature Raman spectra for all of these three sulfates were measured, and crystalline phases were identified at various temperatures.^13−15^

We recently developed a prototype solar-PV cooker based on potassium tetrafluoroborate salt as a heat storage material. We identified that, in general, salts containing tetrahedral molecular anions such as sulfate SO_4_^2–^, tetrafluoroborate BF_4_^–^, molybdate MoO_4_^2–^, and tungstate WO_4_^2–^ are promising materials for latent heat storage applications at a wide range of temperatures. In order to harvest them for thermal energy storage applications, it is essential to conduct detailed thermal analysis over many heating/cooling cycles to check their thermal stabilities.

**Figure 1:**
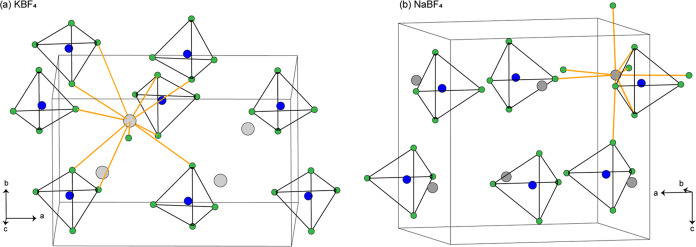
Crystal structures of the ambient temperature phases (a) KBF_4_ (space group *Pnma*, *Z* = 4) and (b) NaBF_4_ (space group *Cmcm*, *Z* = 4). Boron, fluorine, and metal atoms are represented as blue, green, and gray circles, respectively. The tetrahedral environment of B atoms by F atoms is emphasized.

In this article, we will therefore revisit order–disorder transitions in two tetrafluoroborate salts (KBF_4_ and NaBF_4_), as the transition enthalpies of both these salts are high. The room-temperature phase of potassium tetrafluoroborate (KBF_4_) is isostructural with that of potassium tetrachlorate with an orthorhombic space group of *Pnma*.^16,17^ The room-temperature crystalline form of NaBF_4_ is also orthorhombic (space group *Cmcm*) and is isostructural with the room-temperature form of NaClO_4_ (α-CaSO_4_ structure type).^18^ In the RT-phase, the K^+^ ion is coordinated by 10 F^–^ ions at distances between 2.76 and 3.08 Å.^19^ The K^+^ polyhedrons are surrounded by six BF_4_ tetrahedra where they share edges (with three) and corners (with four), as depicted in Figure 1. In the RT-phase of NaBF_4_, the Na^+^ ion is coordinated by 8 F^–^ ions at distances between 2.30 and 2.61 Å. The number of independent F atomic sites for KBF_4_ and NaBF_4_ is two and three, respectively. The BF_4_ tetrahedra are slightly irregular in both structures, and the average B–F distances are around 1.39 Å.

Calorimetric studies of alkali metal tetrafluoroborates have been reported.^20^ KBF_4_ and NaBF_4_ are known to undergo reversible solid–solid phase transformations before their melting points. KBF_4_ undergoes an orthorhombic (*Pnma*) to disordered cubic phase (*Fm*3̅*m*) transition at 285 °C, with Δ*H* = 120 J/g (15.1 kJ/mol). NaBF_4_ is reported to change into a hexagonal structure at ∼230 °C, with Δ*H* = 70 J/g (7.7 kJ/mol). However, the high-temperature crystal structure of NaBF_4_ has not yet been determined. Moreover, the temperature-induced structural changes and the positional parameters as well as thermal displacement parameters are not reported. In situ powder X-ray diffraction measurements at variable temperatures offer a better understanding of thermal expansion coefficients and rotational motions during order–disorder transitions. Moreover, thermal conductivity determines how fast a material conducts heat. However, understanding/measuring thermal conductivity at high temperatures, particularly during phase transitions, is largely unexplored.^21^

The objectives of this research effort were therefore as follows: (i) to conduct detailed thermal analysis using differential scanning calorimetry (DSC) and thermogravimetry (TG) methods to check the thermal stabilities of the materials; (ii) to perform the first in situ synchrotron powder X-ray diffraction study on KBF_4_ and NaBF_4_ to extract the temperature-dependent changes of lattice parameters and thus thermal expansion coefficients of both the RT- and HT-phases; (iii) to determine the high-temperature crystal structure of the disordered phase of NaBF_4_; (iv) to reproduce an experimental phase sequence upon heating, going from fully crystalline to a plastic/rotational phase using molecular dynamics (MD) simulation method; (v) to determine the thermal conductivities of both the compounds in the temperature range of 20–300 °C, which covers their phase transitions.

## 2 Experimental and Computational Methods

### 2.1 Sample

Potassium tetrafluoroborate, CAS 14075-53-7 (99.99% trace metal basis), and sodium tetrafluoroborate, CAS 13755-29-8 (98% purity), were purchased from Sigma-Aldrich and Fluorochem, respectively. The sample bottles were kept in a desiccator and were used without further purification.

### 2.2 Simultaneous Thermal Analysis

Differential scanning calorimetry (DSC) and thermogravimetric analysis (TGA) measurements were performed on a NETZSCH STA 449 F3 instrument by using alumina crucibles with lids. Pure nitrogen was used as a purge gas at a flow rate of 50 mL/min. The samples with masses of about 10–15 mg were heated at 5 K/min to 350 °C. The reference sample in the case of all measurements was an empty crucible.

### 2.3 Thermal Conductivity Measurements

The thermal diffusivity (α) values of NaBF_4_ and KBF_4_ were measured with the laser flash method using a NETZSCH-LFA instrument. Sample disks with 13 mm diameter and ∼1.5 mm thickness were prepared by cold-pressing powders at 5 and 10 ton uniaxial pressure. Before measurement, the pellets were coated using graphite spray. The thermal conductivity was calculated using κ = *C*_p_ × ρ × α, where *C*_p_ is the heat capacity (approximated using the Dulong–Petit value) and ρ is the gravimetric density. The densities of the pellets pressed at 10 tons were near the theoretical limit, with the 5 ton samples having slightly lower densities near 95% of the theoretical. The theoretical densities were used in the calculations for all samples, including the lower crystallographic density for the high-temperature phases.

### 2.4 Variable Temperature Powder X-ray Diffraction Measurements

The samples were contained in 1 mm diameter thin-walled borosilicate glass capillaries that were spun at 919 rpm on the axis of the high-resolution powder diffractometer at beamline ID22 at the European Synchrotron Radiation Facility.^22^ The X-ray wavelength was calibrated as 0.354331(7) Å (35 keV) via a NIST standard 640c Si powder. Diffraction patterns were collected in continuous scanning mode using the 13-channel Si 111 multianalyzer stage at 10 or 20°/min and recording data every 3 or 1.5 ms, respectively. Data were corrected for the effects of axial divergence,^23,24^ and the 13 channels were combined and rebinned into steps of 0.0007°. Heating was via a hot-air blower.

### 2.5 Rietveld Analysis

Rietveld analysis of PXRD patterns was performed using the Topas Academic V7.^25^ Crystal structures of KBF_4_ and NaBF_4_ were refined using orthorhombic *Pnma* and *Cmcm* space groups, respectively, before phase transitions. The scale factor, background parameters, instrumental zero-point, lattice parameters, and peak profile parameters (a full Voigt function was used) were initially refined. To fit the highly anisotropic peak shapes, the Stephens *hkl*-dependent peak shape model was used.^26^ In the final Rietveld refinements, all atom positions were reliably refined without restraints. Neither set of data would give a stable refinement if the occupation factors and thermal parameters were simultaneously refined. The occupation factors for all of the atoms were therefore constrained to achieve charge balance. Isotropic thermal parameters (*B*_iso_) for the individual atoms were refined for the ordered structures. However, for the HT disordered phases, *B*_iso_ for individual atom types yielded high and unreliable values; therefore, an overall common *B*_iso_ for all of the atoms was set and refined. Thermal expansion coefficients were calculated using the PASCAL program.^27^

### 2.6 Computational Methods

Density functional theory (DFT) calculations were performed for structure optimizations and molecular dynamics (MD) simulations, using the Vienna ab initio simulation package VASP,^28^ together with PAW pseudopotentials^29^ that included 7 (3) valence electrons for K/Na/F (B), respectively. Electronic exchange-correlation effects were described with the Perdew–Burke–Ernzerhof (PBE) functional.^30^ Plane wave basis set cutoff was set to *E*_c_ = 30 Ry (408 eV). Brillouin zone sampling for geometry optimizations (MD) used regular grids with density 20/Å^–1^ (the Baldereschi point (1/4,1/4,1/4)). MD simulations were run at 300–600 K in 100 K steps for both compounds and within the NVT and NPT ensembles. The timestep in the MD is d*t* = 2.0 fs. Ideally, the MD supercells of the low-temperature phases can accommodate the high-temperature phases.

For KBF_4_, the low-temperature *Pnma* structure, following a lattice transformation a′ = (−a, 2b, 0), b′ = (a, 2b, 0), and c′ = (0, 0, 2c), results in a (2, 2, 2) supercell of the high-temperature *Fm*3̅*m* structure. The main difference is the γ angle, which is 76.6° in *Pnma* and 90° in *Fm*3̅*m*. In NPT simulations, this supercell (which has 192 atoms and 32 formula units) allows for a direct *Pnma* → *Fm*3̅*m* transition. For NaBF_4_, we performed independent calculations on the *Cmcm* and *P*6_3_/*mmc* phases, using for *Cmcm* a supercell with a′ = (a, b, −2c), b′ = (a, b, 2c), and c′ = (a, −b, 0) (192 atoms) and for *P*6_3_/*mmc* a diagonal (3, 3, 2) supercell (216 atoms).

## 3 Results and Discussion

### 3.1 Thermal Stability

**Figure 2:**
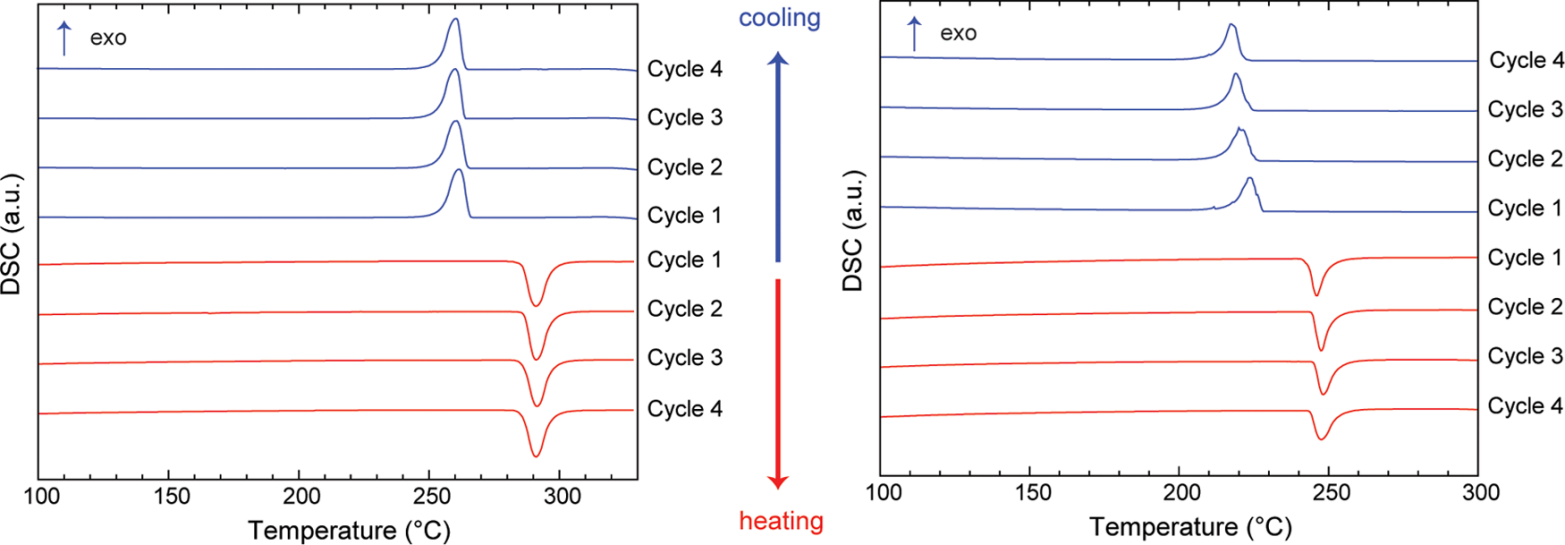
Differential scanning calorimetry (DSC) plots of KBF_4_ (left) and NaBF_4_ (right) over four heating and cooling cycles.

This work was intended to determine the thermal stabilities of the two tetrafluoroborate salts (NaBF_4_ and KBF_4_) by DSC and TGA. Thermal analysis results are shown in Figure 2 and are compiled in Table 1. The DSC curve of KBF_4_ showed one endothermic peak at 291(1) °C, corresponding to *Pnma* → *Fm*3̅*m* transition. On cooling, reversible phase changes occur, giving a sharp exothermic peak, at 260(1) °C. The phase change enthalpy of the KBF_4_ sample was measured to be 110–112 J/g during heating and 117–120 J/g during cooling. The DSC signal of NaBF_4_ was measured between room temperature and 330 °C. During heating, only one endotherm peak at 246(2) °C with a Δ*H*_heating_ of 62–64 J/g was observed. However, on cooling, the transition was obtained at 220(3) °C with a slightly higher latent heat Δ*H*_cooling_ of 72–78 J/g. All of these findings are in good agreement with that reported earlier.^31^ Interestingly, for both phases, Δ*H*_cooling_ > Δ*H*_heating_. We cannot explain the difference in the Δ*H* values for the heating and cooling phases. The integrated areas appear to be slightly larger for the exothermic peaks than the corresponding endothermic ones. However, subsequent heating and cooling cycles gave essentially the same trend. The thermal measurements with the heating–cooling cycles were repeated several times to also check the thermal degradation of the samples over multiple cycles. The residual masses (%) of the samples remain 100(1)% after 4 cycles, which shows the thermal stability of these materials. Unfortunately, due to a limited amount of time and resources, it was not possible to conduct the study more than over a few cycles. However, for heat storage applications, thermal stability tests should be conducted for >1000 cycles in an industrial setting.

**Table 1:** Transition Temperatures, Enthalpy Values, and Residual Masses of KBF_4_ and NaBF_4_ Salts from DSC and TGA Measurements Using Four Heating and Cooling Cycles

	KBF_4_						NaBF_4_				
cycle		onset (°C)	peak (°C)	end-point (°C)	latent heat (J/g)	residual mass (%)	onset (°C)	peak (°C)	end-point (°C)	latent heat (J/g)	residual mass (%)
1	heating	286	291	299	110	100	243	246	250	64	101
	cooling	264	260	254	117	100	227	223	212	72	101
2	heating	286	291	299	112	100	245	248	251	63	100
	cooling	264	261	252	120	100	225	220	217	78	100
3	heating	286	291.4	297	112	99	245	248	253	62	100
	cooling	264	260.1	253.6	119	99	223	219	216	77	100
4	heating	286	290.9	296.8	112	100	244	248	253	62	100
	cooling	263	260.4	253.9	118	100	221	217	214	78	100

### 3.2 Structural Behavior of KBF_4_ at Elevated Temperatures

**Figure 3:**
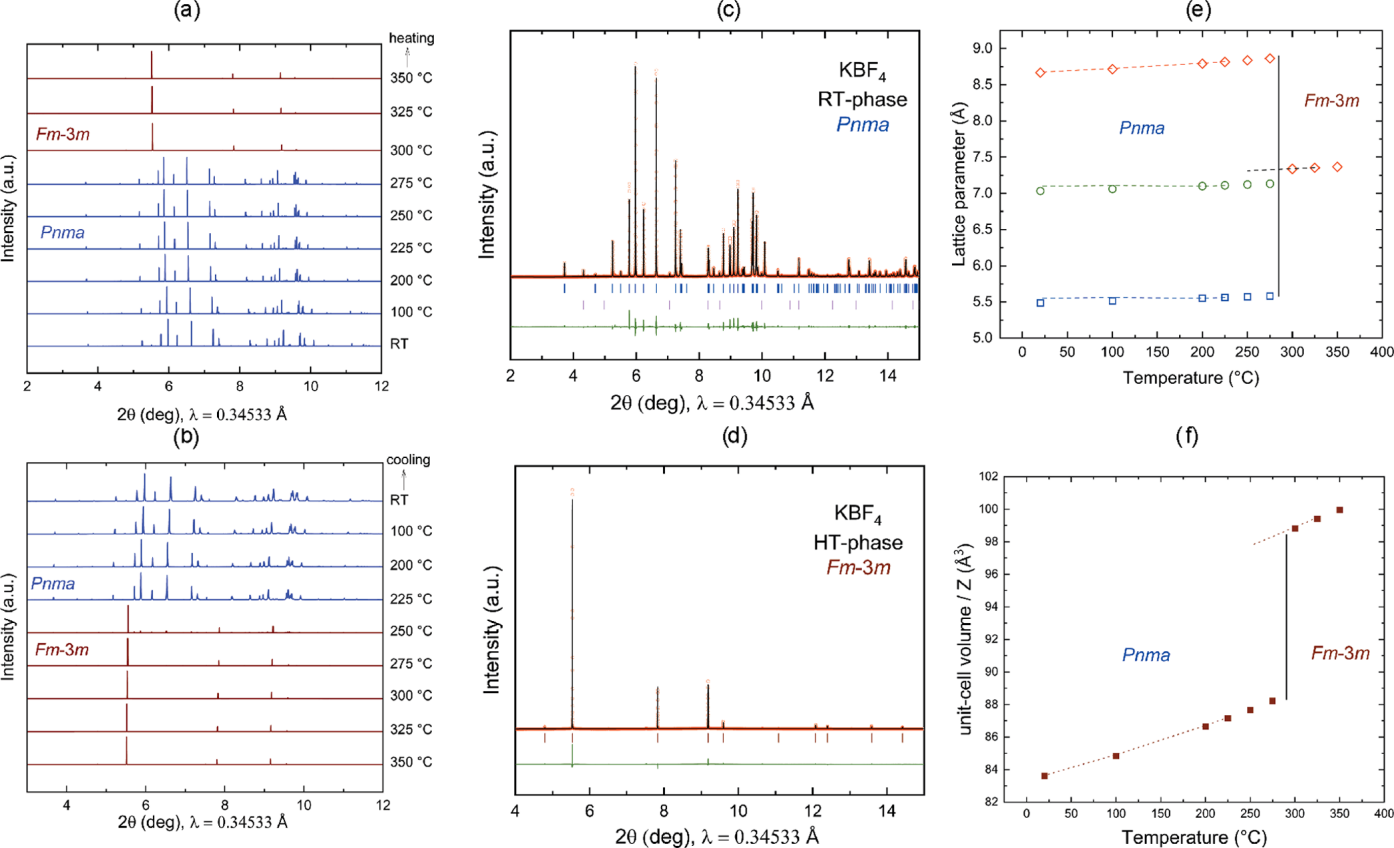
(a, b) Compilation of in situ PXRD patterns of KBF_4_ at variable temperatures (RT–350 °C) showing the transition from RT orthorhombic (*Pnma*) to HT cubic (*Fm*3̅*m*) phase during heating and cooling: blue line, *Pnma* and wine line, *Fm*3̅*m*; (c, d) Rietveld refinement fit of RT- and HT-phases of KBF_4_, experimental (observed) data are shown as red dots, the solid black line shows the calculated profile from the refinements, and the bottom green traces show the residual intensities *I*(obs) – *I*(calc). The simulated Bragg reflections for the phases are given as vertical tick marks (blue, KBF_4_*Pnma* phase; magenta, K_2_SIF_6_ impurity phase); (e, f) temperature dependence of the lattice parameters and unit-cell volume of LT- and HT-KBF_4_ as obtained from synchrotron powder diffraction data; diamond, square, and circular symbols represent lattice parameters *a*, *b*, and *c*, respectively. Corresponding dotted lines represent data from the cooling cycles.

To follow the influence of the thermal treatment, a sample was heated and cooled between RT and 350 °C over multiple heating/cooling cycles. We start the discussion with the results obtained from the first cycle. Diffraction patterns were obtained at 20, 100, 200, and every 25 °C up to 350 °C. The synchrotron powder X-ray diffraction (PXRD) plots are shown in Figure 3a,b. From the XRD traces, the RT orthorhombic (*Pnma*) to HT disordered cubic (*Fm*3̅*m*) phase transition occurs at 300 °C during heating (Figure 3a). Upon cooling, a hysteresis is observed and the HT-phase remains up to 250 °C, and a pure orthorhombic phase is visible only at 225 °C (Figure 3b). The results are consistent with the DSC data. But it should be noted that the transition temperature of a sample may depend not only upon the purity of the sample but also on the size of the crystallites in the powder and the heating rate.

PXRD data taken at 20 °C (first and second heating cycles) were refined using the structural data of the room-temperature phase of KBF_4_ determined by Brunton.^19^ The quality of the Rietveld fits (for cycle 1, *R*_wp_ = 7.6%, for cycle 2, *R*_wp_ = 6.41%) is good. Figure 3c shows the Rietveld fit for cycle 2 data. We also identified a small impurity of the K_2_SiF_6_ phase (<1%). The cell parameters, atomic positions, and isotropic displacement parameters for orthorhombic KBF_4_ are given in Table 2. All our lattice parameters match extremely well with the previous report to within 0.1%, and the overall unit-cell volume differs from the experiment by less than 0.3%.^19^ Atomic parameters also agree closely with those of Brunton as exemplified by bond distances: the B–F distance in the BF_4_^–^ group: 1.365(2)–1.412(2) Å (this work) vs 1.378–1.391 (Brunton); K–F distances: 2.7523(9)–3.0835(4) Å (this work) vs 2.76–3.07 Å (Brunton). PXRD data taken at 300 °C (first heating cycle) were refined using the structural data of the high-temperature phase of KBF_4_ determined by Strømme.^17^ The refinement of the synchrotron data taken at 300 °C showed that RT modification has completely transformed into the HT-phase. The quality of the Rietveld fit (*R*_wp_ = 6.44%) is good, as shown in Figure 3d. The lattice parameters, atomic coordinates, and isotropic displacement parameters for the HT cubic phase of KBF_4_ are given in Table 2.

**Table 2:** Crystallographic Data for RT and HT-Phases of KBF_4_, as Obtained from Rietveld Refinement Results

RT-phase of KBF_4_ (*Pnma*) at 20 °C	
lattice parameter	
*a*	8.66860(3) Å
*b*	5.48559(2) Å
*c*	7.03420(3) Å
*V*	334.493(2) Å^3^

atomic sites and thermal displacement parameters		
	*x*, *y*, *z*	*B*_iso_ (Å^2^)
K (4c)	0.18473(4), 1/4, 0.16140(5)	2.193(8)
B (4c)	0.0653(2), 1/4, 0.6894(2)	2.67(4)
F1 (4c)	0.17810(10), 1/4, 0.55404(10)	2.91(1)
F2 (4c)	–0.08215(10), 1/4, 0.60421(10)	
F3 (8d)	0.07709(6), 0.04297(9), 0.80478(7)	

HT-phase of KBF_4_ (*Fm*3̅*m*) at 300 °C			
lattice parameter			
*a*	7.33877(3) Å		
*V*	395.248(5) Å^3^		
atomic sites and thermal displacement parameters			
	*x*, *y*, *z*	Occ	*B*_iso_ (Å^2^)
K1 (4b)	1/2, 0, 0	0.25	9.27(2)
K2 (4b)	0, 1/2, 0	0.25	
K3 (4b)	0, 0, 1/2	0.25	
K4 (4b)	1/2, 1/2, 1/2	0.25	
B (32f)	–0.0167(5), −0.0167(5), −0.0167(5)	0.125	
F1 (96k)	0.1639(4), −0.0737(3), −0.0737(3)	0.125	
F2 (32f)	–0.1060(8), −0.1060(8), −0.1060(8)	0.125	

The Rietveld refinements were performed at each temperature point (a total of 17 points were measured) on PXRD patterns during heating and cooling. All of the Rietveld fits are available in Figure S1. This allowed us to determine the unit-cell parameters and atomic positions over the temperature change of 20–350 °C. Details of all lattice parameters with reliability parameter (*R*_wp_) are available in Table S1. The lattice parameters and unit-cell volumes of the modifications as a function of the temperature are plotted in Figure 3e,f. The discontinuity at approximately 300 °C is due to *Pnma* → *Fm*3̅*m*. The volume jump is indicative of a first-order transition, as also found for potassium perchlorates.^32^ Throughout the whole temperature range, a positive thermal expansion is found. The orthorhombic cell edge lengths increase anisotropically in a quasilinear manner with *T* up to 275 °C with mean linear expansivities (unit in MK^–1^): α_a_ = 88(3), α_c_ = 54(1), α_b_ = 67.2(6), and α_vol_ = 215(6) and then begin to converge more rapidly as the phase transition to cubic phase is approached. For the HT-phase, α_a_ = 76.7(3) and α_vol_ = 231.4(9).

Table S2 provides changes in B–F and K–F bond distances with temperature. For the *Pnma* phase, there is no appreciable change in the B–F bond distances or F–B–F bond angles as a function of temperature indicating general tetrahedral nature of BF_4_ units. However, BF_4_ tetrahedra become slightly more irregular at higher temperatures. Variations of B–F bond distances and tetrahedral angles are 1.365(2)–1.412(2) Å and 108.20(2)–110.64(1)° at 20 °C, and the corresponding values at 275 °C are 1.324(5)–1.430(3) Å and 104.40(6)–116.8(3)°. Changes in K–F distances (x10) with temperature is also not significant: 2.7523(9)–3.0835(4) at 20 °C and 2.801(2)–3.1528(8) at 275 °C. For the HT *Fm*3̅*m* phase, the variations of bond distances are wider.

The thermal displacement parameters increase steadily across the temperature range of the *Pnma* phase. At room temperature, K/B/F atoms show similar thermal displacement parameters: *B*_K_ = 2.193(8), *B*_B_ = 2.67(4), and *B*_F_ = 2.91(1). However, at 275 °C, the values are highest for the lightest B atoms and lowest for the heaviest K atoms: *B*_K_ = 4.96(2), *B*_B_ = 8.5(1), and *B*_F_ = 6.47(3). For the HT-phase, we were not able to reliably refine the individual thermal displacement parameters for each atom types. So, an overall thermal displacement parameter was set and refined. Temperature-dependent thermal displacement parameters are provided in Table S3.

### 3.3 Structural Behavior of NaBF_4_ at Elevated Temperatures

**Figure 4:**
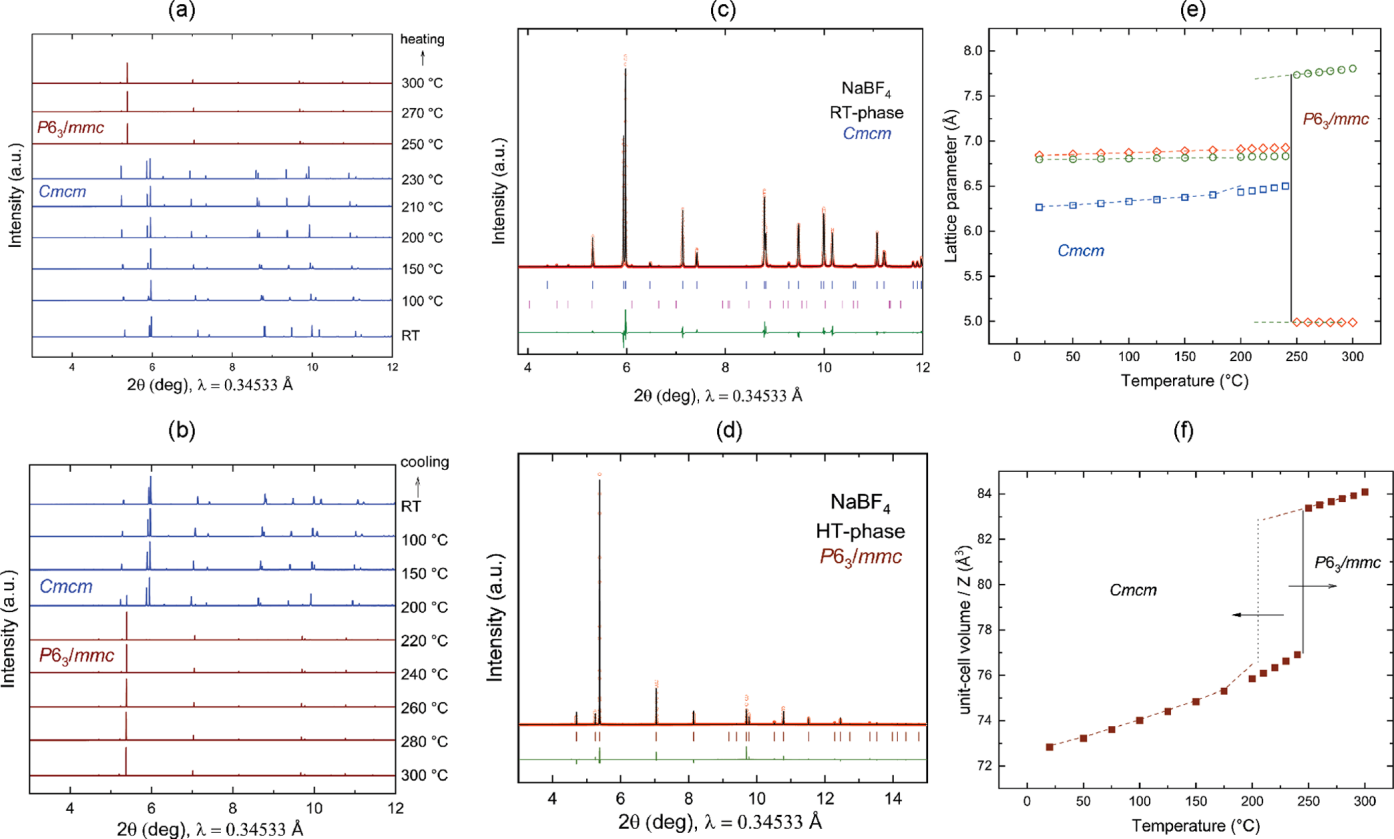
(a, b) Compilation of in situ PXRD patterns of NaBF_4_ at variable temperatures (RT–300 °C) showing the transition from RT orthorhombic (*Cmcm*) to HT hexagonal (*P*6_3_/*mmc*) phase during heating and cooling: blue line, *Cmcm* and wine line, *P*6_3_/*mmc*; (c, d) Rietveld refinement fit of RT- and HT-phases of NaBF_4_, experimental (observed) data are shown as red dots, the solid black line shows the calculated profile from the refinements, and the bottom green traces show the residual intensities *I*(obs) – *I*(calc). The simulated Bragg reflections for the phases are given as vertical tick marks (blue, NaBF_4_*Cmcm* phase; magenta, Na_2_SIF_6_ impurity phase); (e, f) temperature dependence of the lattice parameters and unit-cell volume of LT- and HT-NaBF_4_ as obtained from synchrotron powder diffraction data; diamond, square, and circular symbols represent lattice parameters *a*, *b*, and *c*, respectively. Corresponding dotted lines represent data from cooling cycles.

The synchrotron PXRD data were collected for the NaBF_4_ sample between RT and 300 °C. Diffraction patterns were obtained at 20 °C, every 25 °C in the range of 50–200 °C, and every 10 °C between 200 and 300 °C. The synchrotron powder X-ray diffraction (PXRD) plots are shown in Figure 4a,b. From the XRD traces, the RT orthorhombic (*Cmcm*) to the HT disordered phase transition occurs at 250 °C during heating (Figure 4a). Upon cooling, a hysteresis is observed, the HT-phase remains up to 210 °C, and a pure orthorhombic phase is visible only at 200 °C (Figure 4b).

PXRD data taken at 20 °C (first and second heating cycles) were refined using the structural data of the room-temperature phase of NaBF_4_ determined by Brunton.^18^ The quality of the Rietveld fit (for cycle 1, R_wp_ = 12.01%; for cycle 2, *R*_wp_ = 7.78%) is good. We also identified a small impurity of the Na_2_SiF_6_ phase (<1%). Figure 4c shows the Rietveld fit for cycle 2 data. The unit-cell values, atomic positions, and isotropic displacement parameters for orthorhombic NaBF_4_ are given in Table 3. All our lattice parameters agree well with the previous report to <0.1%, and the overall unit-cell volume differs from the experiment by less than 0.2%.^18^ Atomic parameters also agree closely with those of Brunton as exemplified by the B–F distance in the BF_4_^–^ group: 1.386–1.392 Å (Brunton) to 1.3669(12)–1.4130(13) Å (this work). Na–F distances also agree well with the previous report: 2.3015(5)–2.6110(7) Å (this work) as compared to 2.30–2.61 Å (Brunton).

**Table 3:** Crystallographic Data for RT- and HT-phases of NaBF_4_, as Obtained from Rietveld Refinement of Synchrotron PXRD Data

RT-phase of NaBF_4_ (*Cmcm*) at 20 °C	
lattice parameter	
*a*	6.84242(6) Å
*b*	6.27155(6) Å
*c*	6.79482(6) Å
*V*	291.583(5) Å^3^

atomic sites and thermal displacement parameters			
	*x*, *y*, *z*	occ	*B*_iso_ (Å^2^)
Na (4c)	0, 0.65746(9), 1/4	1	2.03(1)
B (4c)	0, 0.1540(3), 1/4	1	2.04(3)
F1 (8f)	0, 0.29174(7), 0.08546(8)	1	2.43(1)
F2 (8g)	0.16583(7), 0.03244(8), 1/4	1	

HT-phase of NaBF_4_ (*P*6_3_/*mmc*) at 250 °C				
lattice parameter				
*a*		4.98936(2) Å		
*c*		7.73464(4) Å		
*V*		166.748(2) Å^3^		
atomic sites and thermal displacement parameters				
	*x*, *y*, *z*		occ	*B*_iso_ (Å^2^)
Na (2a)	0, 0, 0		1	7.12(3)
B (2c)	1/3, 2/3, 0.25		1	
F1 (12k)	0.2430(1), 0.4859(2), 0.3868(2)		0.333	
F2 (12j)	0.2392(4), 0.3587(2), 1/4		0.333	

The high-temperature phase of NaBF_4_ has not been reported. The synchrotron PXRD pattern taken at 250 °C showed that RT modification has completely transformed into HT-phase. The PXRD pattern at 250 °C can be indexed with a primitive hexagonal unit cell (*a* ≈ 4.99 °A, *c* ≈ 7.73 °A), which pointed to *Z* = 2. Due to the high-resolution PXRD pattern, a space group could be determined to be *P*6_3_/*mmc* from a Pawley refinement. The Rietveld refinement of the HT-phase was attempted with a few published K_2_SO_4_–HT structure types. Two possible orientations of the SO_4_ tetrahedra, i.e., “apex” and “edge” models, for the K_2_SO_4_–HT structure were proposed by Arnold et al.^33^ Rietveld refinement of the 250 °C PXRD pattern yielded a better fit (Figure 4d) with the edge model where three BF_4_ tetrahedra are statistically superimposed. A similar model can also be found in the Na_2_SO_4_–HT-phase.^34^ In our initial model, we set two Na positions (2a and 2d) with site occupancies of 0.5, and we allowed the occupancy values to refine. The occupancy value obtained for the 2a position is close to 1, and therefore, we omitted the 2d position from the model. In the HT-phase model, we set Na at the 2a position, along with other atoms at their respective sites: B (2c), F1 (12k), and F2 (12j). In the HT-phase, the BF_4_ tetrahedra are disordered, and 12 partly occupied F atom positions (occ = 1/3) are associated with three differently oriented BF_4_ groups. The quality of the Rietveld fit using this model is good with a *R*_wp_ = 8.31%. The structural details for the HT hexagonal phase of NaBF_4_ are given in Table 3.

The lattice parameters and unit-cell volumes of both the modifications as a function of the temperature are given in Figure 4e,f. Details of all lattice parameters with reliability parameter (*R*_wp_) are available in Table S4. All of the Rietveld fits are available in Figure S2. The discontinuity at approximately 250 °C is due to *Cmcm* → *P*6_3_/*mmc*. The volume jump is indicative of a first-order transition. For the *Cmcm* phase, throughout the whole temperature range, a positive thermal expansion is found. The orthorhombic cell edge lengths increase anisotropically in a quasi-linear manner with *T* up to 240 °C with mean linear expansivities (unit in MK^–1^): α_a_ = 56.4(3), α_b_ = 165(7), α_c_ = 24.9(6), and α_vol_ = 253(9). In the disordered HT-phase, both the Na–F distances (2.2747(11)–2.4962(2) Å) and B–F distances of (1.3148(13)–1.3639(19) Å.) are somewhat shorter than the average bond distances observed in the RT-phase. The variations in B–F and K–F distances with temperature are provided in Table S5.

For the HT-phase, an overall thermal expansion has been observed with α_vol_ = 165.8(9) MK^–1^ for the temperature range of 250–300 °C. However, a small negative thermal expansion is observed along the *a*–*b* plane (see Table S6). This observation can be visualized easily by following the 100 reflection. With the increase of the temperature, the reflection moves to lower *d*-spacing values. Thermal displacement parameters increase steadily across the temperature range of the *Cmcm* phase. Variations of thermal displacement parameters are provided in Table S7.

### 3.4 Structural Stability over Multiple Heating/Cooling Cycles

**Figure 5:**
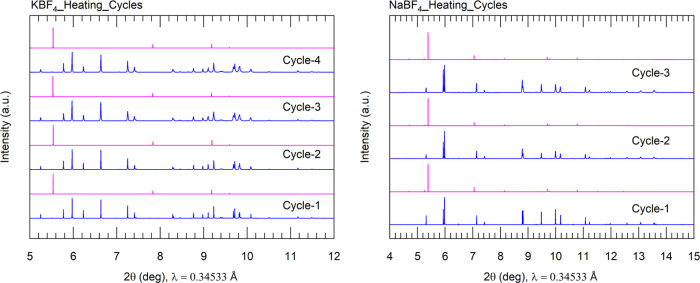
Powder X-ray diffraction patterns before and after phase transitions are shown for KBF_4_ (left) and NaBF_4_ (right).

To check the structural stability of NaBF_4_ and KBF_4_, VT-PXRD measurements were repeated for both samples at all the temperature points. PXRD patterns at a specific temperature from cycle 2 were refined using the same corresponding input files from cycle-1 experiment. The results obtained from the second cycle experiments are consistent with the first cycle. Temperature-dependent lattice parameters are compiled in Tables S8 and S9. The reliability parameters for the second-cycle measurements are in general better (lower *R*_wp_). Lattice parameters from both cycles are individually plotted in Figures S3 and S4 for a direct comparison of structural changes. Apparently, no significant difference is observed across the two data sets. We also managed to conduct additional continuous temperature cycle measurements for both the samples. The PXRD patterns before and after phase transitions for multiple cycles are listed in Figure 5.

### 3.5 Thermal Conductivity Measurements

**Figure 6:**
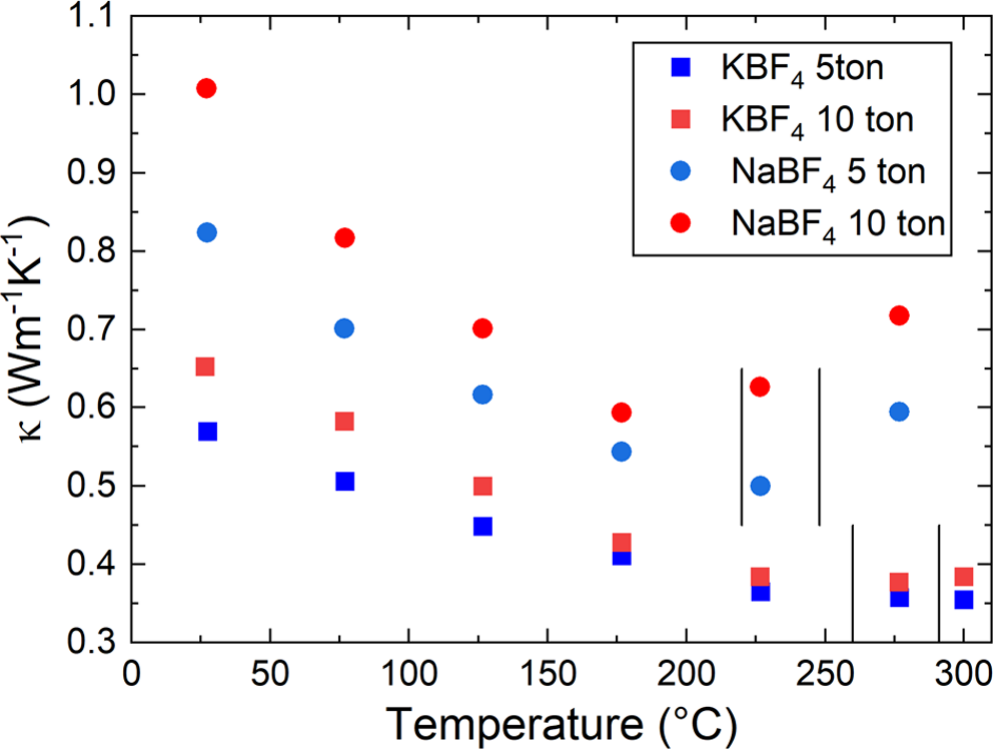
Variation of thermal conductivity of KBF_4_ and NaBF_4_ during heating to 300 °C.

The thermal conductivity (κ) of the pressed disks of NaBF_4_ and KBF_4_ is shown in Figure 6. Vertical lines in Figure 6 represent the region of the structural phase transition. Pressing at 10 tons leads to slightly larger κ than 5 tons, consistent with the higher experimental densities. NaBF_4_ has a higher κ than KBF_4_, as expected based on its lower average atomic mass. At room temperature, the measured values are 0.8–1.0 W m^–1^K^–1^ for NaBF_4_ and 0.55–0.65 W m^–1^K^–1^ for KBF_4_. Below the phase transition, all samples have a temperature dependence broadly in line with Umklapp phonon scattering (κ ∼ 1/*T*). This is typical for crystalline materials. For both compositions, thermal diffusivity (α) values are larger above the phase transition. For KBF_4_, the decrease in the crystallographic density largely offsets the increase in α, leading to a minimal change in κ. For NaBF_4_, the increase in α is much more substantial, leading to a significant increase in κ above the phase transition. The reason for this increase is unclear but is consistent with the increase to a higher symmetry structure, potentially removing some low-energy vibrational modes that contribute to the low κ in the low-temperature phase.

### 3.6 Elucidation of Structure through MD Simulation

#### 3.6.1 DFT

Structural parameters were obtained from geometry optimization of the starting form (*Pnma*, *Z* = 4 for KBF_4_ and *Cmcm*, *Z* = 4 for NaBF_4_) at ambient pressure. The unit cell determined from the calculation (see Table S10) is larger than the values obtained from the experimental reports, which suggests that the DFT method, we used, underestimates the effects of dispersion for the crystal structures at ambient pressure. For KBF_4_, all of our calculated lattice parameters agree with the experiment to within 2.3%, and the overall unit-cell volume differs from the experiment by 6.4%. For NaBF_4_, all of our calculated lattice parameters and the overall unit-cell volume are larger from the experiment to within 1.5% and by less than 4%, respectively.

#### 3.6.2 MD Simulations

**Figure 7:**
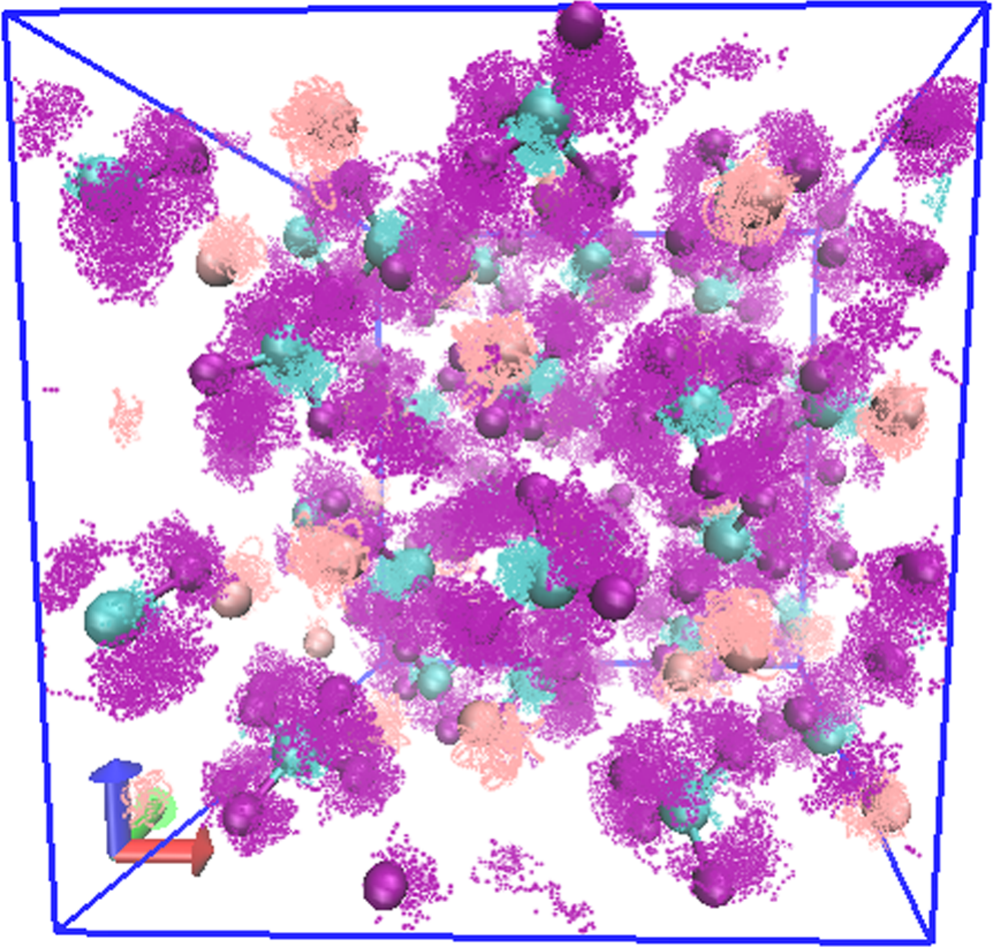
NVT *T* = 600 K trajectory. Orange/cyan/purple spheres denote K/B/F atoms. Supercell is indicated. Complete trajectory is shown.

Figure 7 shows the full trajectory of KBF_4_ at *T* = 600 K visualizing the pronounced motion of F atoms within the solid K/B sublattice. While neither K nor B atoms move away from their lattice sites, F atoms are much more mobile. However, the trajectories of F atoms on adjacent molecules do not overlap, and F atoms are not diffusive. They remain attached to B atoms and form BF_4_^–^ anions at all times, but the anions change orientation rapidly throughout the simulations. In Figure S5, we show the F atom density distributions at *T* = 300/600 K, which show that the BF_4_ units do not rotate freely but have preferred locations for the F atoms (at least within NVT-MD), while below we discuss their mean square displacement to quantify their motion. For KBF_4_, the known relation between the *Pnma* and *Fm*3̅*m* structures allows tracking of the phase transition directly via the lattice evolution in NPT-MD.

**Figure 8:**
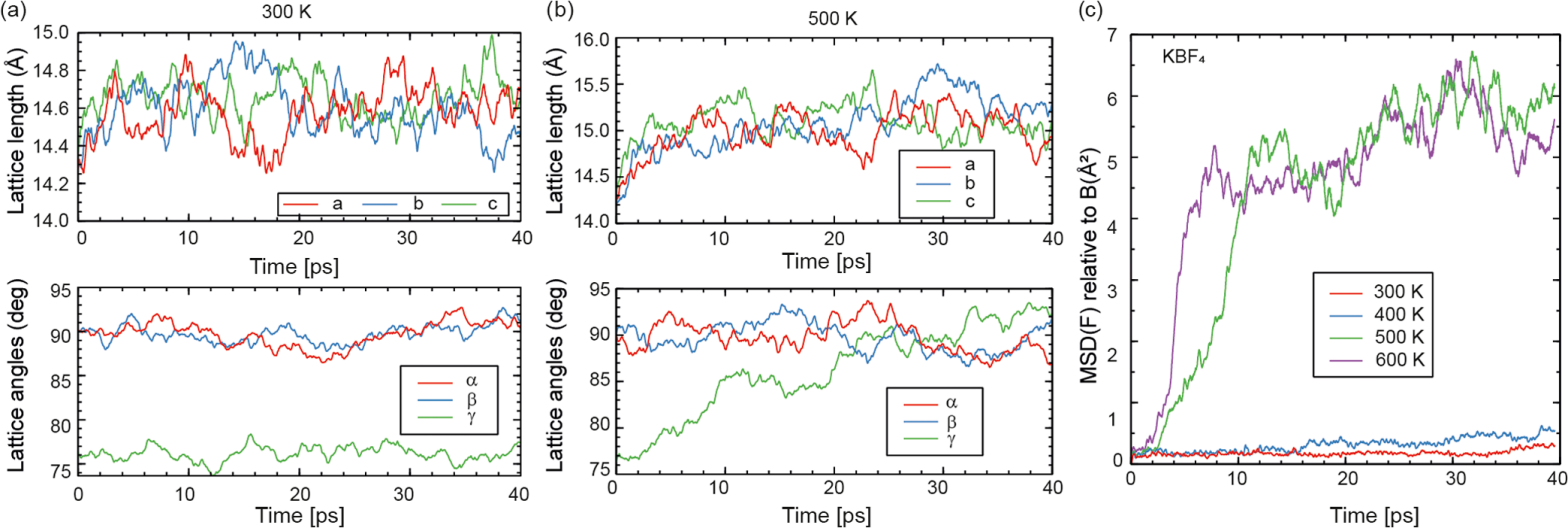
(a, b) Lattice vector lengths and angles during NPT runs at 300 K and 500 K; (c) MSD for NPT runs at *T* = 300–600 K.

Figure 8a,b compares the supercell lattice lengths and angles at *T* = 300 and 500 K. The lattice lengths, within fluctuations, remain constant and equal following equilibration periods to account for thermal expansion. The lattice angles remain constant at 300 K, but at 500 K, the γ angle increases to 90°, which marks the transition to the cubic phase. For NaBF_4_, the supercells remain stable throughout the simulations, but the local atomic motion reveals the transition to the plastic phase, see below.

To quantify the motion of atoms, we plot in Figure 8c the mean square displacement (MSD) of the F atoms, relative to the B atom they are bonded to, defined as MSD_F–B_(*t*) = 1/*N*∑_*i*_(*r*_F_*i*_–B_*i*__(*t*) – *r*_F_*i*_–B_*i*__(0))^2^. For KBF_4_, there is a qualitative difference between the 300/400 and 500/600 K results. At *T* = 300/400 K, the MSD follows what is expected for solids: as the F atoms jitter around their equilibrium lattice positions, total displacements are small and constant over time. At *T* = 500/600 K, the fluorine atoms move much farther, but the MSD ultimately plateaus around 5–6 Å^2^. For BF_4_ molecules that reorient or rotate freely, the expected MSD is MSD(F) = 2 × *r*_BF_^2^ = 4.1 Å^2^; this uses the ground-state B–F bond length of *r*_BF_ = 1.425 Å and should be higher at elevated temperatures. For NaBF_4_, a similar picture holds: (see Figure S6) in the *Cmcm* structure, the rotational state is activated around 400 K, while in the *P*6_3_/*mmc* structure already at 300 K, the BF_4_ molecules are rotational; but note that the structure itself is expected to be metastable at that temperature, so some disagreement with the experimental temperature scale is expected. Figure S7 shows averaged lattice lengths extracted from the HT-MD simulations that were projected back onto the primitive *P*6_3_*/mmc* unit cell. The unit-cell parameters for the HT-phase of NaBF_4_ calculated from MD simulation are very close (within typical DFT uncertainty) to the experimental lattice constants. Moreover, we indeed see a negative thermal expansion along the *a*–*b* plane with an overall thermal expansion of unit-cell volume. This result also supports our experimental finding (discussed in Section 3.3).

We also analyzed the partial distribution functions (PDFs) of the different atom types (see Figure S8) for the different phases. These confirmed that BF_4_ molecules remained intact and the long-range order of the K/Na–B sublattice remained throughout the simulations.

## 4 Conclusions

Structural changes of sodium and potassium tetrafluoroborate were studied using variable temperature powder X-ray diffraction measurements within the temperature range of 20–300 °C for NaBF_4_ and 20–350 °C for KBF_4_, respectively. Structural phase transitions are consistent with the differential scanning calorimetry data. KBF_4_ undergoes a reversible phase transition from *Pnma* to *Fm*3̅*m* at 290 °C (Δ*H* = 117–120 J/g). Order–disorder transition for NaBF_4_ occurs at 246 °C (Δ*H* = 64 J/g). The high-temperature phase of NaBF_4_ was determined from the synchrotron powder X-ray data at 250 °C. The HT-phase belongs to *the P*6_3_/*mmc* space group and is apparently very similar to HT-K_2_SO_4_ structure type. Lattice constants obtained from MD simulation are very close (within typical DFT uncertainty) to the experimental values. From the high-precision unit-cell parameters from the Rietveld analysis of synchrotron powder XRD data, thermal expansion coefficients were determined. Anisotropic thermal expansion coefficients are observed for both the RT and HT-phases of NaBF_4_ and KBF_4_. Interestingly, the HT-phase of NaBF_4_ shows negative thermal expansion along the *a-b* plane. The contraction along the *a-b* plane is also observed in the MD simulation. Thermal conductivities (κ) of both the samples were measured at room temperature; κ = 0.8–1.0 W m^–1^K^–1^ for NaBF_4_ and κ = 0.55–0.65 W m^–1^K^–1^ for KBF_4_. Below the phase transition, κ for both materials show temperature dependence broadly in line with Umklapp phonon scattering (κ ∼ 1/*T*). Both NaBF_4_ and KBF_4_ show very good structural and thermal stability over a few heating–cooling cycling. This paper highlights the importance of a systematic and detailed structural and thermal investigation on solid–solid phase-change materials using a combined experimental and theoretical approach. We believe that this work should bring significant interest to explore inorganic salts containing tetrahedral molecular anions such as sulfates, molybdates, tetrafluoroborates, and tungstates for thermal energy storage applications.

## Supporting Information Available

Tables: Variation of unit-cell parameters, bond lengths (B–F and Na/K–F distances) and thermal displacement parameters of KBF_4_ and NaBF_4_ during heating and cooling at a set of temperatures across phase transitions; data are obtained from Rietveld refinement of the synchrotron PXRD patterns. Crystallographic data of KBF_4_ and NaBF_4_ at ambient pressure, as obtained from the current DFT study. Principal coefficients of thermal expansion and corresponding principal axes for NaBF_4_–HT-phase. Figures: Rietveld refinement plots of synchrotron powder diffraction patterns of KBF_4_ and NaBF_4_ at elevated temperatures. Comparison of the lattice parameters and unit-cell volume of LT- and HT-KBF_4_ and NaBF_4_ from cycle 1 and cycle 2 experiments; values are obtained from Rietveld refinement of synchrotron powder diffraction data. F atom real-space distribution throughout KBF_4_-*Pnma* using NVT-MD. MSD(t) for F atoms relative to their bonded B atoms from HT-MD NPT runs at various temperatures. Averaged lattice lengths extracted from the HT-MD simulations and projected back onto the primitive *P*6_3_/*mmc* unit cell. Partial distribution functions (PDF’s) for NPT simulations of KBF_4_ and NaBF_4_ at a set of temperatures (PDF)
