# Supplementary material for: Revisiting Solid–Solid Phase Transitions in Sodium and Potassium Tetrafluoroborate for Thermal Energy Storage
Source: Chem Mater. 2024 Jan 31;36(3):1238–48. doi: 10.1021/acs.chemmater.3c02039 (PMC10870698; doi:10.1021/acs.chemmater.3c02039)

SUPPORTING INFORMATION

Revisiting Solid-solid Phase Transitions in Sodium and Potassium Tetrafluoroborate for Thermal Energy Storage

Sumit Konar[a], Gertruda Zieniute[a], Elliot Lascelles[a], Beth Wild[a], Andreas Hermann[b], Yi Wang[c], Robert J. Quinn[d], Jan-Willem G. Bos[e], Andrew Fitch[f]

[a]Joseph Banks Laboratories, School of Chemistry, University of Lincoln, Lincoln, LN6 7DL, United Kingdom

[b]Centre for Science at Extreme Conditions and SUPA, School of Physics and Astronomy, The University of Edinburgh, Edinburgh, EH9 3FD, United Kingdom

[c]School of Chemical Engineering, University of Birmingham, Birmingham, B15 2TT, United Kingdom

[d]Institute of Chemical Sciences, School of Engineering & Physical Sciences, Heriot-Watt University, Edinburgh, EH14 4AS, United Kingdom

[e]EaStCHEM School of Chemistry, University of St Andrews, North Haugh, St Andrews, KY16 9ST, United Kingdom

[f]European Synchrotron Radiation Facility, 71 avenue des Martyrs, Grenoble 38000, France

***E-mail:** **skonar@lincoln.ac.uk**

Table S1. For cycle 1, variation of unit cell parameters of KBF_4 (solid powdered sample loaded in a borosilicate capillary) during heating and cooling in the temperature range of 20 – 350 °C. Data are obtained from Rietveld refinement of the synchrotron PXRD patterns. Values in parentheses are estimated standard deviations.

	Temp (°C)	*a* (Å)	*b* (Å)	c (Å)	V (Å^3)	Rwp
	Pnma					
heating	20 °C	8.66631(2)	5.486077(14)	7.034675(17)	334.457(1)	8.1
	100 °C	8.71493(3)	5.51407(2)	7.06189(2)	339.360(2)	8.7
	200 °C	8.79175(4)	5.55147(2)	7.10029(3)	346.546(3)	8.8
	225 °C	8.81514(3)	5.561351(18)	7.11103(2)	348.619(2)	8.8
	250 °C	8.83894(3)	5.570915(16)	7.12157(2)	350.680(2)	9
	275 °C	8.86514(3)	5.580700(18)	7.13277(2)	352.884(2)	9.5
	Fm-3*m*					
	300 °C	7.339007(19)			395.286(3)	9.9
	325 °C	7.35336(4)			397.611(6)	10.6
	350 °C	7.36720(3)			399.860(6)	10.9
cooling	325 °C	7.35535(3)			397.934(6)	11.3
	300 °C	7.34090(3)			395.593(5)	11.4
	275 °C	7.32647(3)			393.265(5)	11.5
	250 °C	7.31179(6)			390.90(1)	14.6
	Pnma					
	225 °C	8.82155(8)	5.56207(5)	7.11116(6)	348.916(6)	8.5
	200 °C	8.79871(17)	5.55195(11)	7.10002(13)	346.84(1)	8.4
	100 °C	8.72455(11)	5.51489(7)	7.05965(9)	339.675(7)	7.9
	20 °C	8.67441(10)	5.48701(7)	7.03059(8)	334.632(7)	7.7

Table S2. For Cycle 1, variation of B – F and K – F bond distances of KBF_4 during heating and cooling in the temperature range of 20 – 350 °C. Data are obtained from Rietveld refinement of the synchrotron PXRD patterns. Values in parentheses are estimated standard deviations.

		Distances (Å)			
		RT-phase *Pnma*			
		B1 – F1	B1 – F2	B1 – F3	K – F distances range
heating	20 °C	1.3649(21)	1.3939(20)	1.4251(14)	2.7461(9) – 3.0762(4)
	100 °C	1.3591(29)	1.3950(29)	1.4277(18)	2.7655(12) – 3.0978(6)
	200 °C	1.3383(38)	1.3947(38)	1.4305(23)	2.7839(13) – 3.1289(6)
	225 °C	1.3358(38)	1.3901(38)	1.4303(23)	2.7923(13) – 3.1366(6)
	250 °C	1.3280(38)	1.3906(38)	1.4291(23)	2.7963(14) – 3.1454(7)
	275 °C	1.3225(46)	1.3892(47)	1.4300(28)	2.8017(16) – 3.1528(8)
		HT-phase *Fm*-3*m*			
		B – F distances range			K – F distances range
	300 °C	1.1249(112) – 1.6354(72)			2.5898(37) – 3.0936(95)
	325 °C	1.092(129) – 1.6614(82)			2.5942(44) – 3.0987(10)
	350 °C	1.1655(145) – 1.6191(90)			2.6035(52) – 3.1015(125)
Cooling	325 °C	1.1757(149)- 1.6066(96)			2.6011(51) – 3.0966(125)
	300 °C	1.1866(143) – 1.5955(96)			2.6025(51) – 3.0896(117)
	275 °C	1.1919(142) – 1.5905(92)			2.5946(51) – 3.0832(117)
	250 °C	1.2286(264) – 1.5396(171)			2.6009(88) – 3.0795(219)
		RT-phase *Pnma*			
		B1 – F1	B1 – F2	B1 – F3	
	225 °C	1.3256(61)	1.4933(63)	1.3615(34)	2.7822(16) – 3.1473(8)
	200 °C	1.3319(53)	1.4817(54)	1.3716(30)	2.7772(15) – 3.1373(8)
	100 °C	1.3472(37)	1.4638(37)	1.3850(22)	2.7626(13) – 3.1041(6)
	20 °C	1.3639(29)	1.4445(29)	1.3887(17)	2.7517(12) – 3.0814(5)

Table S3. For Cycle 1, variation of thermal displacement parameters of KBF_4 during heating and cooling in the temperature range of 20 – 350 °C. Data are obtained from Rietveld refinement of the synchrotron PXRD patterns. Values in parentheses are estimated standard deviations.

	B (Å^2)		
	B_K	B_B	B_F
	RT-phase *Pnma*		
20 °C	2.240 (8)	2.58 (4)	2.89 (1)
100 °C	2.95 (1)	4.01 (2)	3.78 (2)
200 °C	3.95 (2)	6.38 (9)	5.16 (2)
225 °C	4.28 (2)	6.82 (9)	5.63 (2)
250 °C	4.55 (2)	7.6 (1)	6.06 (2)
275 °C	4.96 (2)	8.5 (1)	6.47 (3)
	HT-phase *Fm*-3*m*		
300 °C	9.35(4)		
325 °C	9.97(4)		
350 °C	9.94(4)		
325 °C	9.35(4)		
300 °C	8.65(4)		
275 °C	8.30(4)		
250 °C	mixed phase, not so reliable		
	RT-phase *Pnma*		
225 °C	4.75 (3)	16.7 (2)	6.05 (3)
200 °C	4.31 (2)	12.0 (2)	5.55 (3)
100 °C	3.08 (1)	6.62 (9)	3.92 (2)
20 °C	2.29 (1)	4.20 (7)	2.96 (1)

Table S4. For cycle 1, variation of unit cell parameters $NaBF_4$ (solid powdered sample loaded in a borosilicate capillary) during heating and cooling in the temperature range of 20 – 300 °C. Data are obtained from Rietveld refinement of the synchrotron PXRD patterns. Values in parentheses are estimated standard deviations.

	Temp (°C)	*a* (Å)	*b* (Å)	c (Å)	V (Å^3)	Rwp
heating	20 °C	6.84175(2)	6.266154(19)	6.79562(2)	291.338(2)	12.8
	50 °C	6.85304(5)	6.28638(5)	6.79933(5)	292.921(4)	13.8
	75 °C	6.86288(7)	6.30660(7)	6.80343(7)	294.462(5)	12.5
	100 °C	6.87224(9)	6.32843(9)	6.80741(9)	296.057(7)	12.5
	125 °C	6.88116(12)	6.35061(11)	6.81131(11)	297.651(9)	12.5
	150 °C	6.89041(13)	6.37466(12)	6.81543(12)	299.36(1)	12
	175 °C	6.90019(9)	6.40146(9)	6.81951(8)	301.227(7)	13.9
	200 °C	6.91059(3)	6.43287(3)	6.82428(3)	303.373(2)	12.9
	210 °C	6.91509(2)	6.44775(2)	6.82636(2)	304.365(2)	13.1
	220 °C	6.91923(2)	6.463434(19)	6.828199(19)	305.371(2)	13.4
	230 °C	6.923556(18)	6.480912(17)	6.830293(18)	306.482(1)	13.4
	240 °C	6.92766(3)	6.49950(2)	6.83231(2)	307.634(2)	15.4
	250 °C	4.989553(13)		7.73498(3)	166.768(1)	14.8
	260 °C	4.989049(13)		7.74923(3)	167.042	14.9
	270 °C	4.988605(15)		7.76356(3)	167.321(1)	15.1
	280 °C	4.988191(15)		7.77770(3)	167.598(1)	14.2
	290 °C	4.987871(15)		7.79204(3)	167.885(1)	14.3
	300 °C	4.98732(4)		7.80569(7)	168.142(3)	14.2
cooling	290 °C	4.987651(15)		7.79504(3)	167.935(1)	14.4
	280 °C	4.987864(15)		7.78149(3)	167.657(1)	14.5
	270 °C	4.988100(15)		7.76745(3)	167.371(1)	14.7
	260 °C	4.988425(15)		7.75408(3)	167.104(1)	14.8
	250 °C	4.988731(15)		7.74011(3)	166.824(1)	14.9
	240 °C	4.988947(15)		7.72663(3)	166.548(1)	15.1
	230 °C	4.98906(3)		7.71293(6)	166.260(3)	16
	220 °C	4.98937(4)		7.69947(8)	165.990(3)	15.4
	210 °C	6.91614(9)	6.45170(8)	6.82631(9)	304.596(7)	13.7
	200 °C	6.91165(9)	6.43747(9)	6.82412(9)	303.629(7)	15
	175 °C	6.90112(9)	6.40632(9)	6.81966(9)	301.502(7)	11.2
	150 °C	6.89052(7)	6.37948(7)	6.81502(7)	299.574(5)	10.5
	100 °C	6.87045(8)	6.33362(8)	6.80632(8)	296.176(6)	10.1
	75 °C	6.86092(8)	6.31267(8)	6.80222(8)	294.609(6)	10
	50 °C	6.85171(8)	6.29213(8)	6.79844(8)	293.093(6)	10
	20 °C	6.84240(8)	6.27151(8)	6.79464(8)	291.573(6)	9.9

Table S5. For Cycle 1, variation of B – F and Na – F bond distances of $NaBF_4$ during heating and cooling in the temperature range of 20 – 300 °C. Data are obtained from Rietveld refinement of the synchrotron PXRD patterns. Values in parentheses are estimated standard deviations.

		Distances (Å)		
		B1 – F1	B1 – F2	Na – F distances range
heating	20 °C	1.3985(17)	1.4089(16)	2.2989(8)– 2.618(1))
	50 °C	1.4122(25)	1.3986(23)	2.3048(10) – 2.6388(12)
	75 °C	1.3877(24)	1.4280(24)	2.3009(9) – 2.6396(12)
	100 °C	1.3710(24)	1.4472(25)	2.3034(10) – 2.6531(13)
	125 °C	1.3551(27)	1.4647(29)	2.3017(10) – 2.6653(13)
	150 °C	1.3521(27)	1.4614(29)	2.3036(10) – 2.6747(13)
	175 °C	1.3847(32)	1.4152(33)	2.3057(12) – 2.6922(16)
	200 °C	1.3581(28)	1.4437(30)	2.3124(11) – 2.7004(16)
	210 °C	1.3823(29)	1.3980(29)	2.3214(12) – 2.7177(16)
	220 °C	1.3899(34)	1.3875(32)	2.3250(12) – 2.7246(16)
	230 °C	1.3893(38)	1.3782(36)	2.3290(13) – 2.7285(17)
		B – F distances range		Na – F distances range
	240 °C			
	250 °C	1.3158(19) – 1.3674(23)		2.2782(16) – 2.4897(12)
	260 °C	1.3128(21) – 1.3699(31)		2.2805(18) – 2.492(1)
	270 °C	1.3217(22) – 1.3672(32)		2.2769(12) – 2.4971(10)
	280 °C	1.3208(22) – 1.3666(26)		2.7282(18) – 2.5013(10)
	290 °C	1.3237(21) – 1.3651(34)		2.2765(18) – 2.5072(10)
	300 °C	1.3151(20) – 1.3667(32)		2.2811(12) – 2.5084(10)
Cooling	290 °C	1.313(2) – 1.3658(21)		2.2825(12) – 2.5058(23)
	280 °C	1.3149(21) – 1.3654(34)		2.2802(18) – 2.5022(10)
	270 °C	1.3211(22) – 1.3674(26)		2.2755(10) – 2.4975(23)
	260 °C	1.3307(22) – 1.3642(34)		2.2682(12) – 2.4966(23)
	250 °C	1.3276(21) – 1.3666(36)		2.2681(18) – 2.4902(21)
	240 °C	1.3338(22) – 1.3637(32)		2.2646(12) – 2.4886(10)
	230 °C	1.3336(21) – 1.3659(26)		2.2624(18) – 2.4840(21)
	220 °C			
	210 °C			
		B1 – F1	B1 – F2	
	200 °C	1.4611(37)	1.2942(29)	2.3289(14) – 2.7040(18)
	175 °C	1.4208(26)	1.3305(23)	2.3266(10) – 2.6866(12)
	150 °C	1.4090(21)	1.3471(19)	2.3208(8) – 2.6723(11)
	100 °C	1.3962(17)	1.3693(16)	2.3134(8) – 2.6481(9)
	75 °C	1.3943(17)	1.3761(16)	2.3100(7) – 2.6366(9)
	50 °C	1.3962(17)	1.3773(16)	2.3080(7) – 2.6251(9)
	20 °C	1.3972(13)	1.3798(13)	2.3062(7) – 2.6142(9)

Table S6 Principal coefficients of thermal expansion and corresponding principal axes for $NaBF_4$-HT phase.

			Directions		
Axes	α (MK^{-1})	σα(MK^{-1})	*a*	*b*	*c*
X1	-8.656	0.2209	0.9391	-0.3437	-0.0
X2	-8.656	0.2209	0.5907	0.8069	-0.0
X3	182.4169	0.7229	-0.0	-0.0	1.0
V	165.7913	0.9307			

Table S7. For Cycle 1, variation of thermal displacement parameters of $NaBF_4$ during heating and cooling in the temperature range of 20 – 300 °C. Data are obtained from Rietveld refinement of the synchrotron PXRD patterns. Values in parentheses are estimated standard deviations.

	Cycle-1			
		Na	B	F
1	20 °C	1.98 (2)	3.18 (6)	2.06 (1)
2	50 °C	2.37 (2)	4.66 (8)	2.34 (2)
3	75 °C	2.58(2)	4.12(8)	2.45 (2)
4	100 °C	2.84(2)	4.61(9)	2.65 (2)
5	125 °C	3.03 (3)	5.4(1)	2.85(2)
6	150 °C	3.29(3)	5.93(1)	3.25(2)
7	175 °C	3.87(4)	4.61(1)	3.62(3)
8	200 °C	4.71(4)	4.41 (9)	4.11 (2)
9	210 °C	4.54(3)	5.0(1)	4.44(3)
10	220 °C	4.67(3)	6.9(1)	4.74(3)
11	230 °C	4.69(4)	6.7(1)	4.98(3)
12	240 °C	Mixed phase, so not reliable		
13	250 °C	6.91(4)		
14	260 °C	6.96(4)		
15	270 °C	7.22(5)		
16	280 °C	7.40(5)		
17	290 °C	7.53(5)		
18	300 °C	7.68(5)		
19	290 °C	7.35(5)		
20	280 °C	7.22(5)		
21	270 °C	7.15(5)		
22	260 °C	7.13(5)		
23	250 °C	6.90(5)		
24	240 °C	6.85(5)		
25	230 °C	6.78(5)		
26	220 °C	6.51(5)		
27	210 °C	Mixed phase, so not reliable		
28	200 °C	4.13 (4)	4.9 (1)	5.08 (3)
29	175 °C	3.51 (3)	4.05 (7)	4.53 (2)
30	150 °C	3.16 (2)	3.57 (6)	4.05 (2)
31	100 °C	2.61 (2)	2.63 (5)	3.26 (2)
32	75 °C	2.34 (2)	2.27 (4)	2.92 (1)
33	50 °C	2.08 (1)	2.02 (3)	2.60 (1)
34	20 °C	1.85 (1)	1.77 (3)	2.311 (9)

Table S8. For cycle 2, variation of unit cell parameters KBF_4 (solid powdered sample loaded in a borosilicate capillary) during heating and cooling in the temperature range of 20 – 350 °C. Data are obtained from Rietveld refinement of the synchrotron PXRD patterns. Values in parentheses are estimated standard deviations.

	Temp (°C)	*a* (Å)	*b* (Å)	*c* (Å)	*V* (Å^3)	Rwp
	Pnma					
heating	20 °C	8.66866(4)	5.48558(3)	7.03421(3)	334.495(3)	7.8
	100 °C	8.71446(7)	5.51219(4)	7.06085(5)	339.174(5)	9.6
	200 °C	8.78764(8)	5.54893(5)	7.09860(6)	346.142(5)	9.2
	225 °C	8.80949(7)	5.55861(5)	7.10867(6)	348.101(5)	9.4
	250 °C	8.83415(8)	5.56863(5)	7.11941(6)	350.233(5)	9.4
	275 °C	8.85925(7)	5.57807(5)	7.12995(6)	352.344(5)	9.4
	Fm-3*m*					
	300 °C	7.335583(18)			394.734(3)	11.2
	325 °C	7.349767(18)			397.028(3)	12.3
	350 °C	7.363644(16)			399.281(3)	13.4
cooling	325 °C	7.35518(2)			397.905(3)	14.3
	300 °C	7.34334(3)			395.988(5)	14.2
	275 °C	7.33009(3)			393.847(5)	14.4
	Pnma					
	250 °C	8.85273(17)	5.57403(11)	7.12456(14)	351.56(1)	13.3
	225 °C	8.82751(12)	5.56420(7)	7.11367(9)	349.414(6)	9.6
	200 °C	8.80432(8)	5.55434(5)	7.10292(6)	347.348(5)	9.5
	100 °C	8.72822(9)	5.51650(6)	7.06268(8)	340.062(6)	9.4
	20 °C	8.67347(8)	5.48670(5)	7.03224(7)	334.656(6)	9.1

Table S9. For cycle 2 variation of unit cell parameters $NaBF_4$ (solid powdered sample loaded in a borosilicate capillary) during heating and cooling cycle 1 in the temperature range of 20 – 300 °C. Data are obtained from Rietveld refinement of the synchrotron PXRD patterns. Values in parentheses are estimated standard deviations.

	Temp (°C)	*a* (Å)	*b* (Å)	c (Å)	V (Å^3)	Rwp
heating	50 °C	6.85205(7)	6.29059(7)	6.79888(7)	293.055(5)	10.1
	75 °C	6.86122(6)	6.30929(6)	6.80305(6)	294.500(5)	10.2
	100 °C	6.87086(6)	6.32982(6)	6.80734(6)	296.060(5)	10.3
	125 °C	6.88061(6)	6.35173(6)	6.81163(6)	297.694(5)	10.3
	150 °C	6.89054(6)	6.37563(6)	6.81591(6)	299.433(5)	10.6
	175 °C	6.90066(6)	6.40242(6)	6.82019(6)	301.322(5)	12.2
	200 °C	6.91127(7)	6.43364(7)	6.82468(6)	303.457(5)	12.2
	210 °C	6.91587(7)	6.44888(7)	6.82661(7)	304.464(5)	12.5
	230 °C	6.92428(7)	6.48175(7)	6.83038(7)	306.558(6)	12.9
	240 °C	6.92838(7)	6.50107(7)	6.83241(7)	307.744(6)	13
	250 °C	4.98880(3)		7.73743(5)	166.770(2)	14
	260 °C	4.98810(5)		7.75112(10)	167.019(4)	14
	270 °C	4.98780(5)		7.76524(9)	167.303(4)	14.3
	280 °C	4.98738(5)		7.77938(9)	167.579(4)	13.4
	290 °C	4.98709(5)		7.79373(8)	167.869(4)	13.6
	300 °C	4.98677(5)		7.80769(8)	168.148(4)	13.6
cooling	290 °C	4.987079(16)		7.79674(3)	167.933(1)	13.8
	280 °C	4.987314(15)		7.78331(3)	167.660(1)	13.9
	270 °C	4.987662(15)		7.76957(3)	167.387(1)	14.1
	260 °C	4.987955(14)		7.75574(3)	167.109(1)	14.1
	250 °C	4.988291(15)		7.74206(3)	166.836(1)	14.2
	240 °C	4.988575(17)		7.72845(3)	166.562(1)	14.3
	230 °C	4.98872(5)		7.71488(8)	166.280(3)	15.2
	220 °C	4.98905(5)		7.70148(8)	166.012(4)	14.1
	210 °C	6.91672(12)	6.45092(13)	6.82662(11)	304.599(9)	14.1
	200 °C	6.91211(12)	6.43899(12)	6.82404(12)	303.718(9)	12.9
	175 °C	6.90123(10)	6.40824(10)	6.81946(10)	301.589(8)	10.5
	150 °C	6.89020(9)	6.38004(9)	6.81480(9)	299.577(7)	10
	125 °C	6.88024(10)	6.35766(10)	6.81018(10)	297.893(8)	10.9
	100 °C	6.87050(10)	6.33609(10)	6.80605(10)	296.282(8)	9.3
	75 °C	6.86080(10)	6.31521(10)	6.80191(10)	294.709(8)	8.8
	50 °C	6.85173(10)	6.29515(10)	6.79804(10)	293.217(8)	9.2
	20 °C	6.84229(10)	6.27359(9)	6.79428(9)	291.649(7)	9.1

Table S10: Crystallographic data of KBF_4 and $NaBF_4$ at ambient pressure, as obtained from current DFT study.

KBF_4 (*Pnma*)			$NaBF_4$ (C*mcm*)		
Lattice parameter			Lattice parameter		
a		8.8275 Å	*a*		6.9168 Å
b		5.6121 Å	*b*		6.3373 Å
c		7.1844 Å	*c*		6.8993 Å
V		334.46	V		302.42
Atomic sites (x, y, z)			Atomic sites (x, y, z)		
K	4c	0.18436, ¼,0.15966	Na	4c	0, 0.65245, ¼
B	4c	0.06177, ¼, 0.68854	B	4c	0, 0.15957, ¼
F1	4c	0.17899, ¼, 0.55241	F1	8f	0, 0.2921, 0.08268
F2	4c	-1/12, ¼, 0.60347	F2	8g	0.16748, 0.02867, ¼
F3	8d	0.07708, 0.04250, 0.80391			

Figure S1 Rietveld refinement plots of synchrotron powder diffraction patterns of KBF_4 at elevated temperatures.

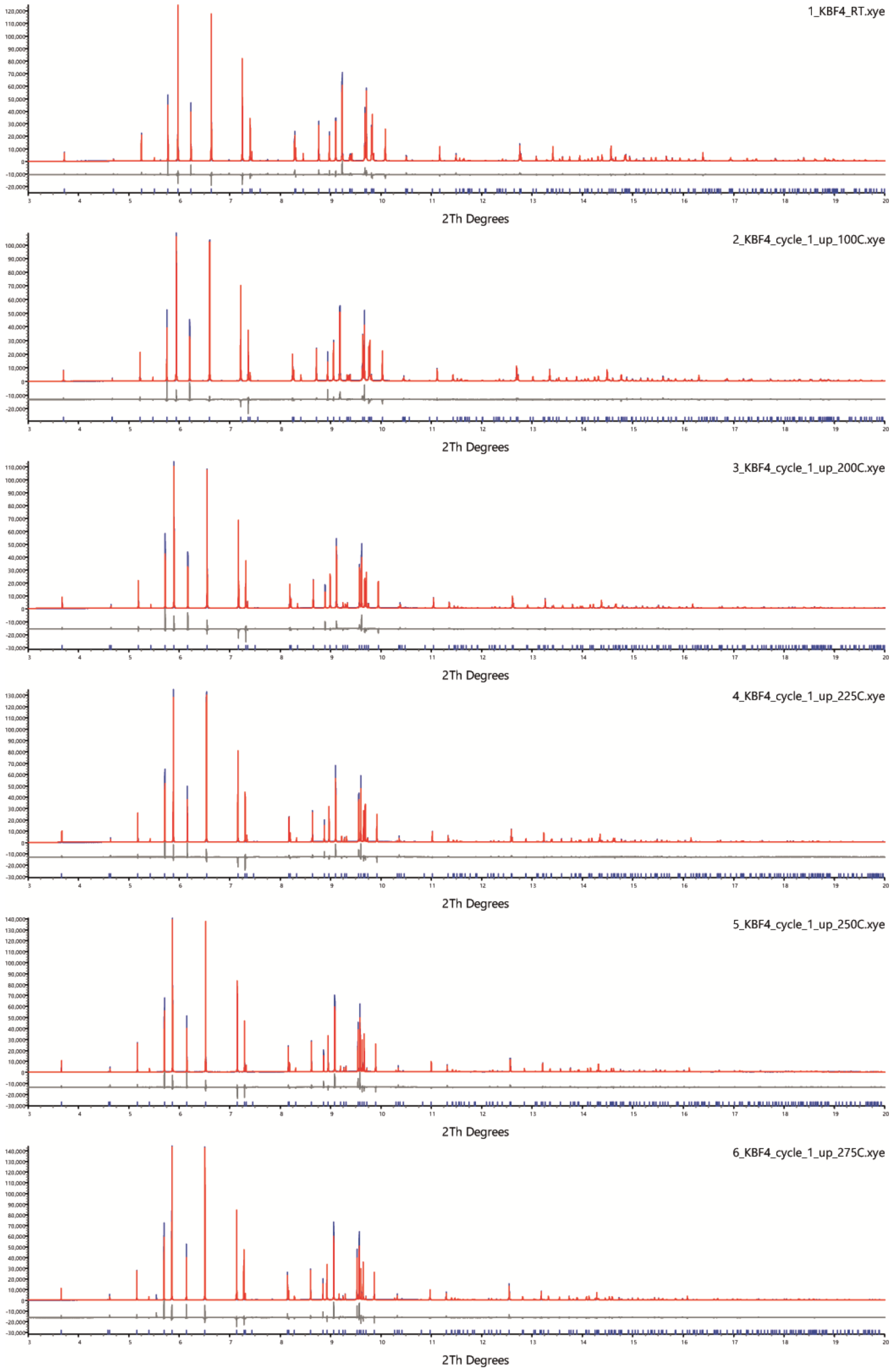

Figure S1 Rietveld refinement plots of Synchrotron powder diffraction patterns of KBF_4 at elevated temperatures (continued).

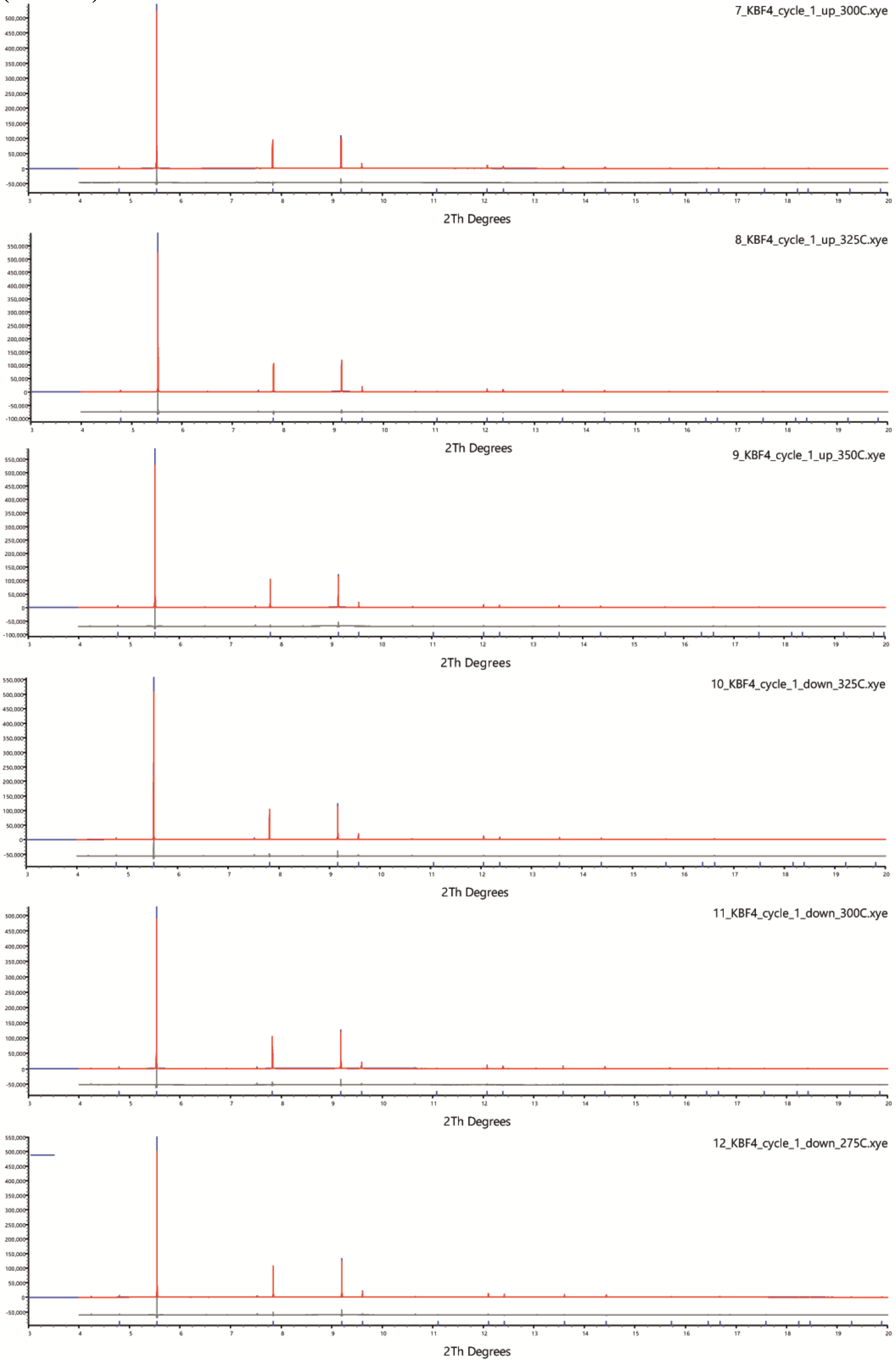

Figure S1 Rietveld refinement plots of synchrotron powder diffraction patterns of KBF_4 at elevated temperatures.

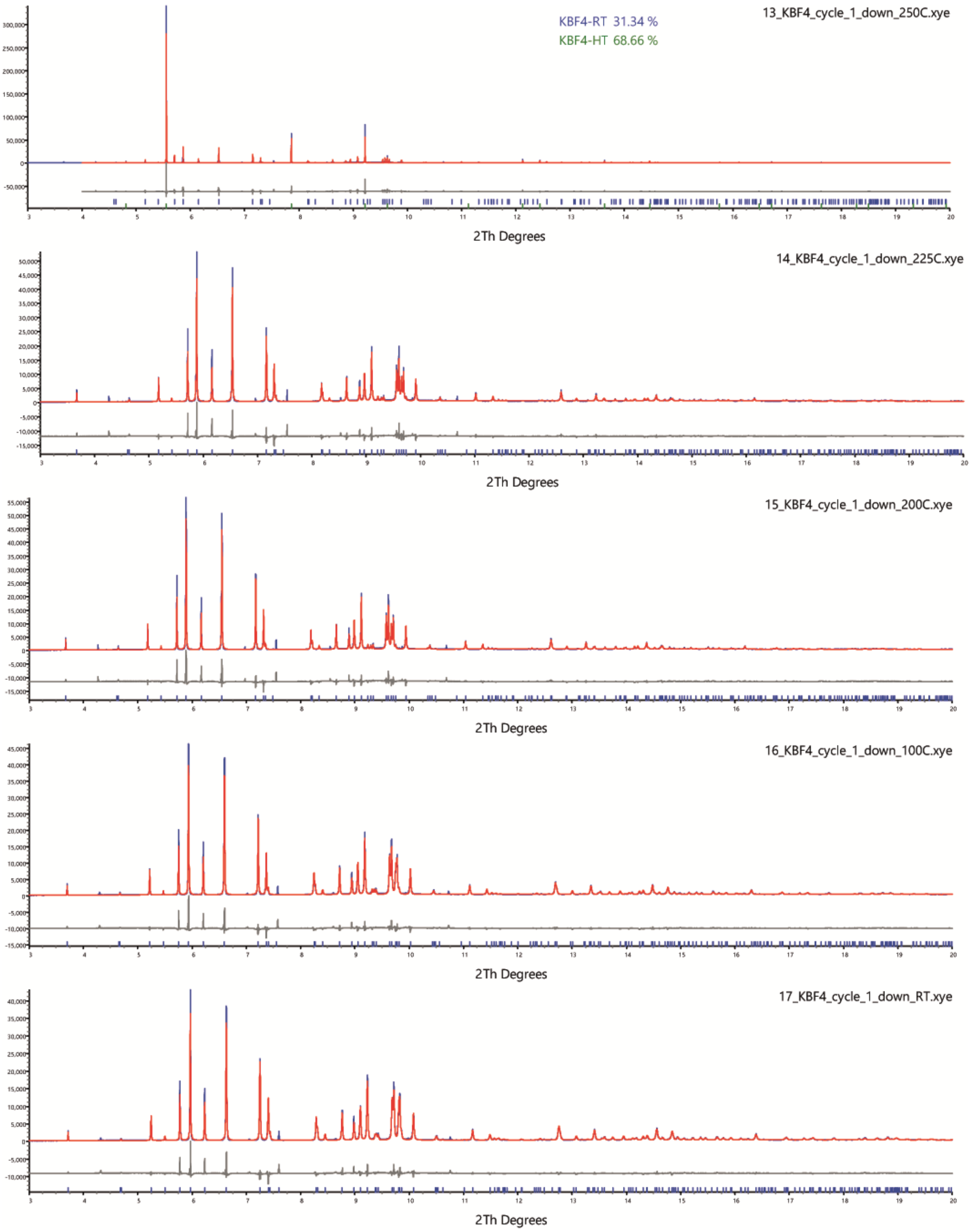

Figure S2 Rietveld refinement plots of synchrotron powder diffraction patterns of $NaBF_4$ at elevated temperatures.

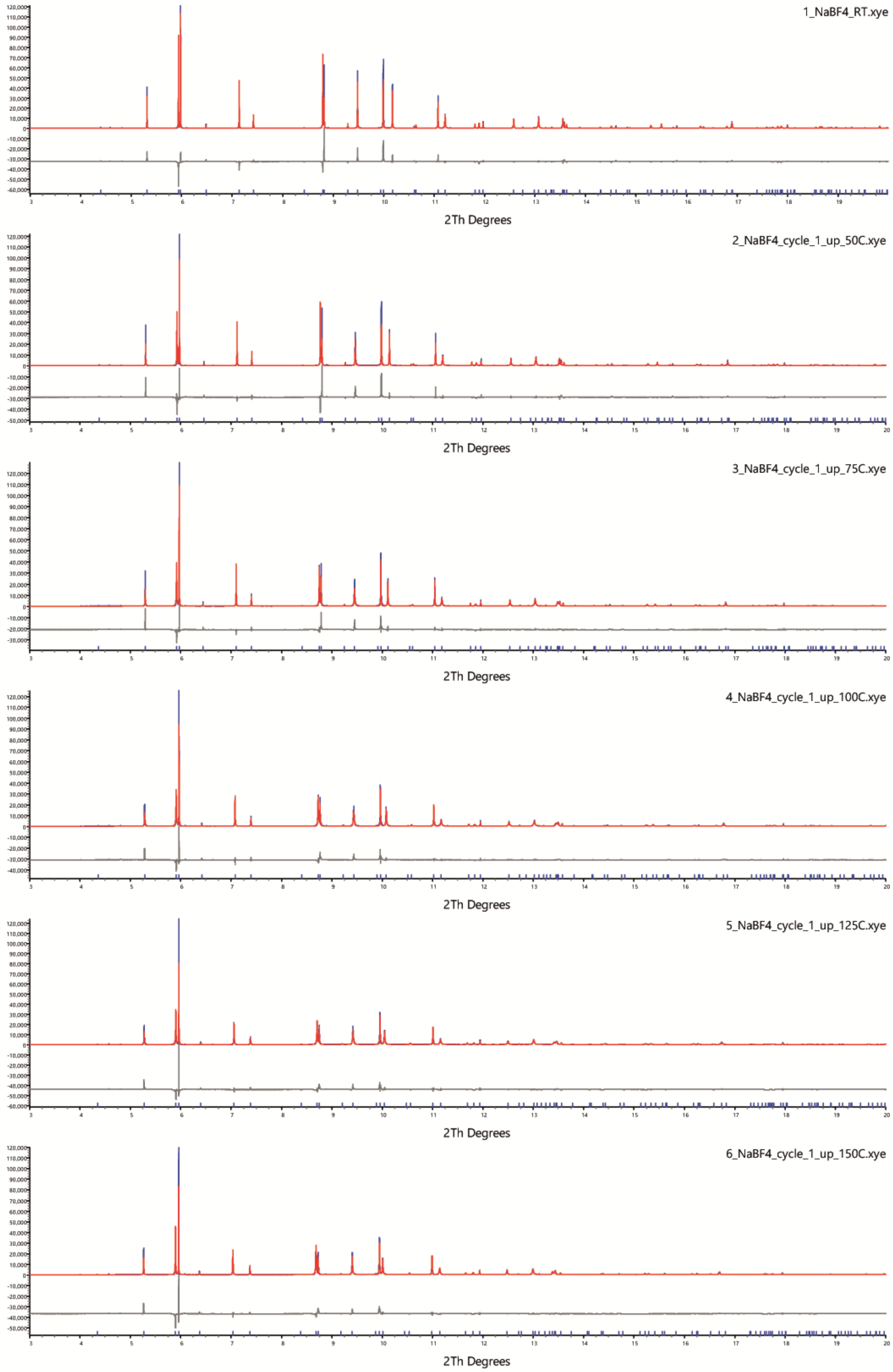

Figure S2 Rietveld refinement plots of synchrotron powder diffraction patterns of $NaBF_4$ at elevated temperatures (continued).

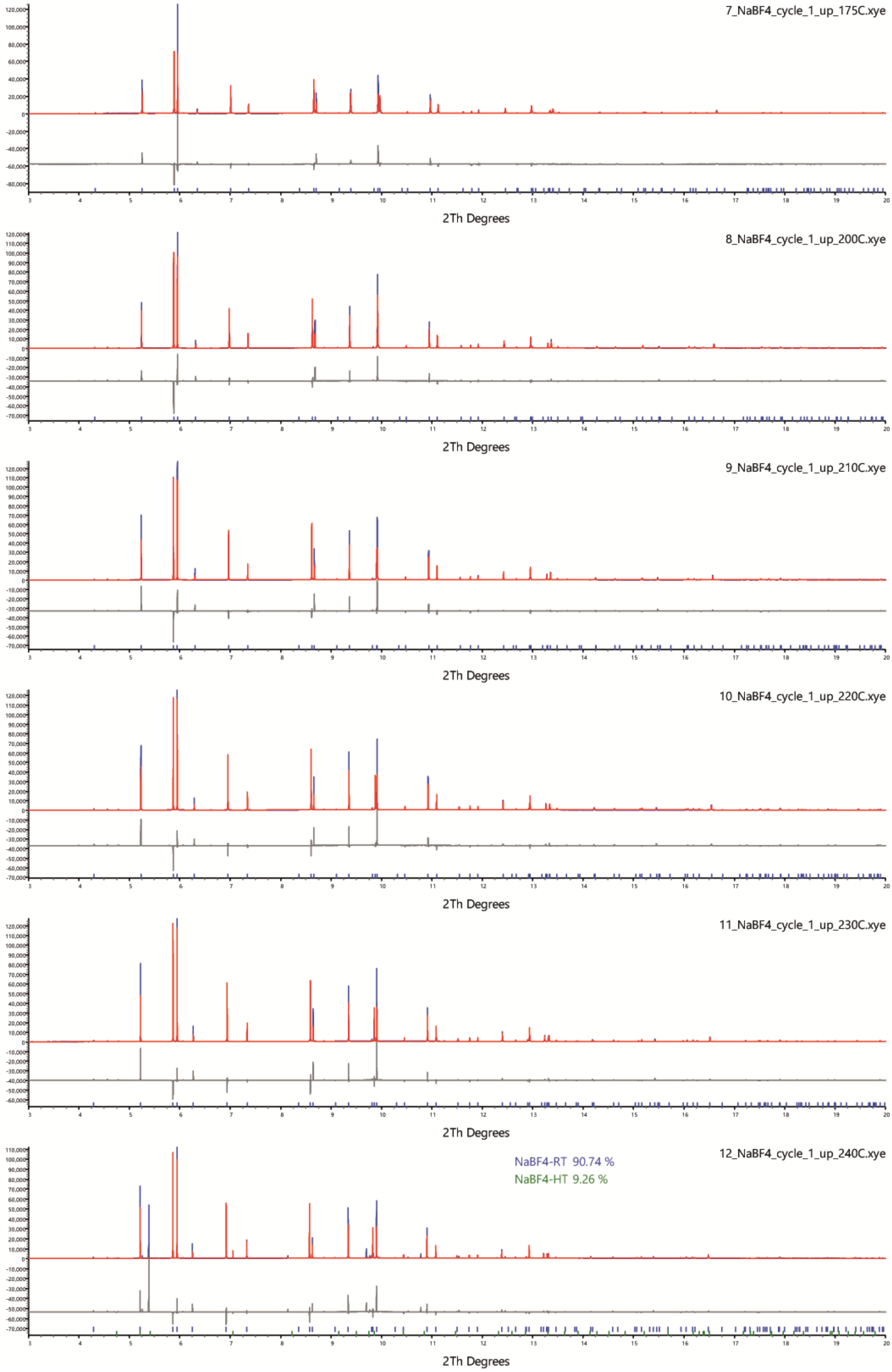

Figure S2 Rietveld refinement plots of synchrotron powder diffraction patterns of $NaBF_4$ at elevated temperatures (continued).

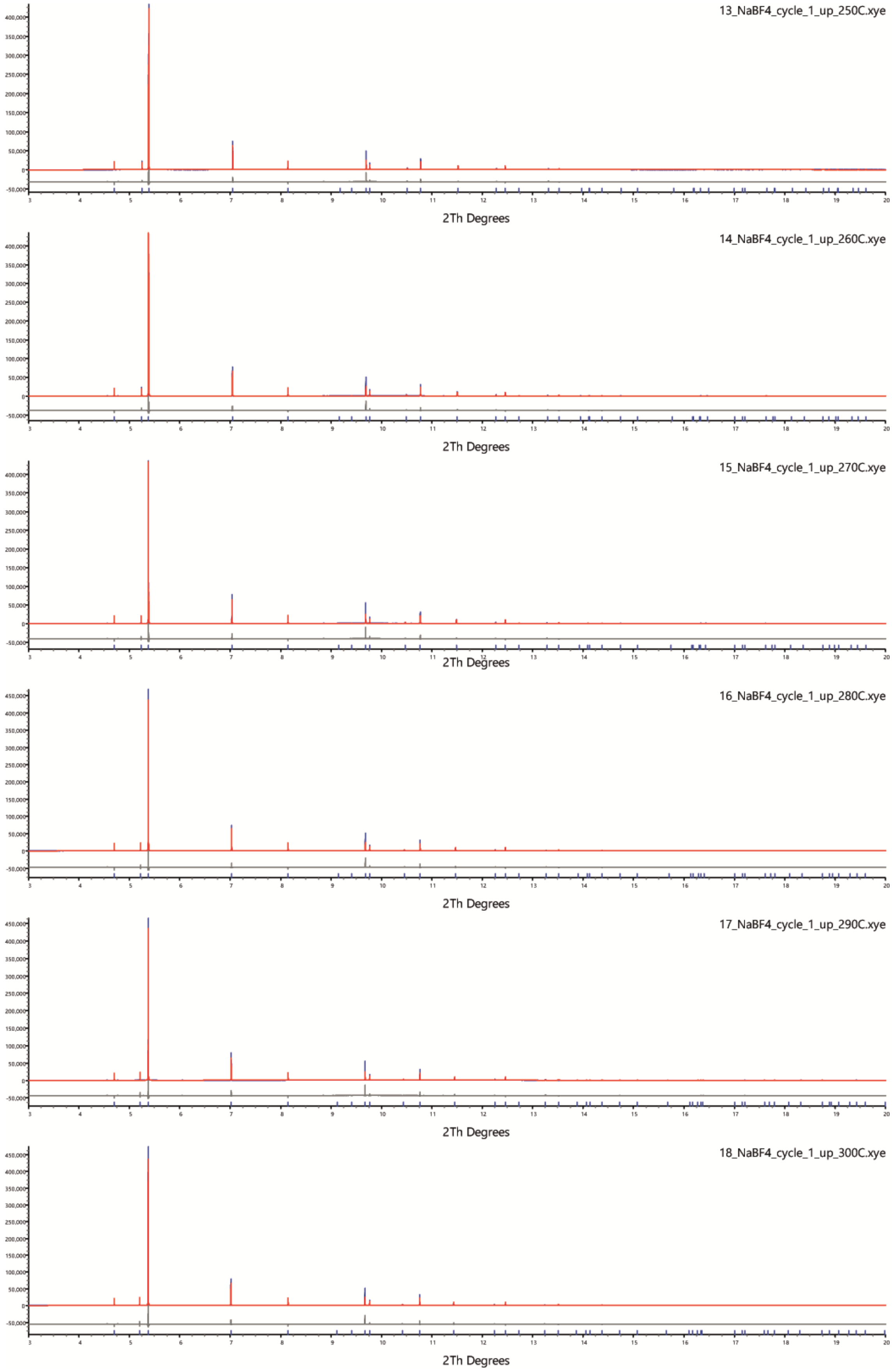

Figure S2 Rietveld refinement plots of synchrotron powder diffraction patterns of $NaBF_4$ at elevated temperatures (continued).

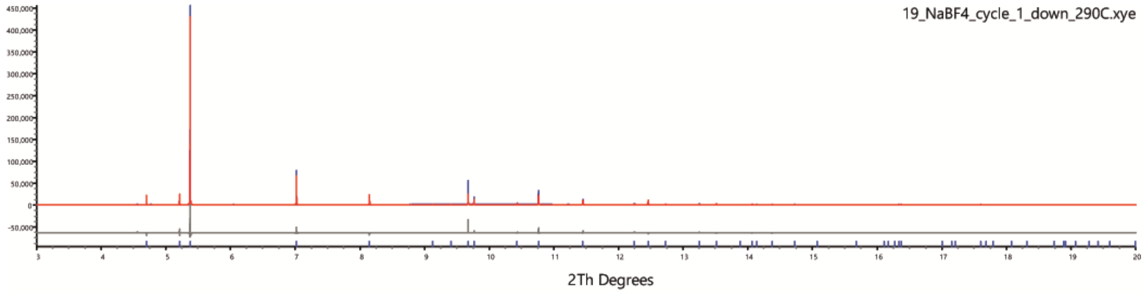

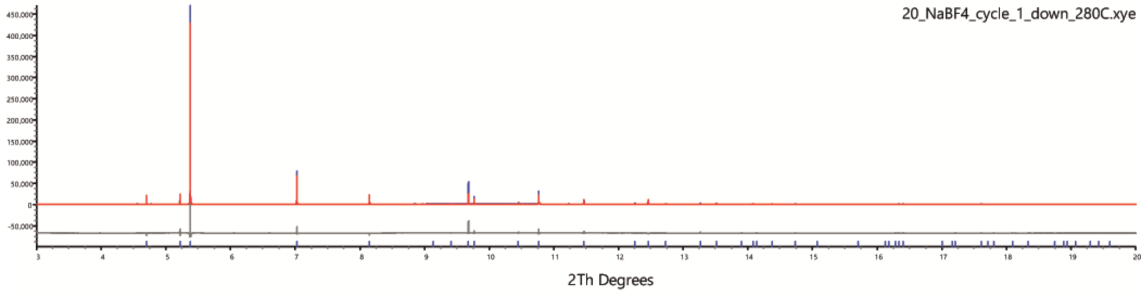

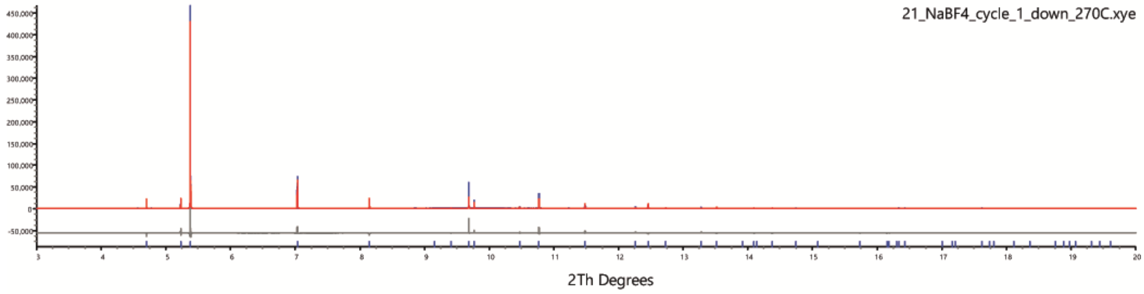

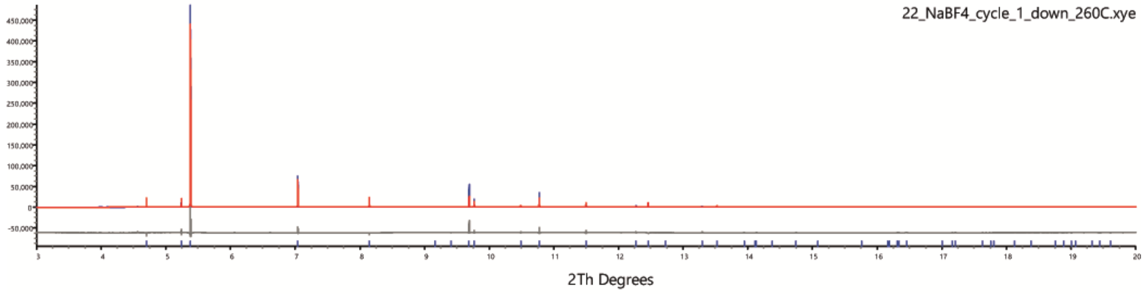

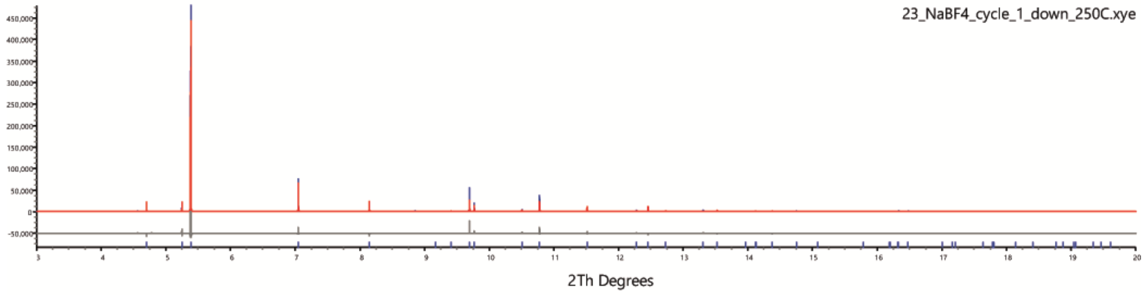

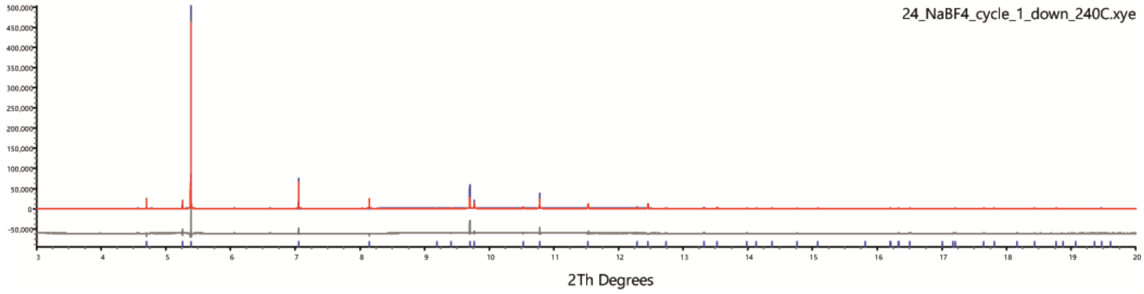

Figure S2 Rietveld refinement plots of synchrotron powder diffraction patterns of $NaBF_4$ at elevated temperatures (continued).

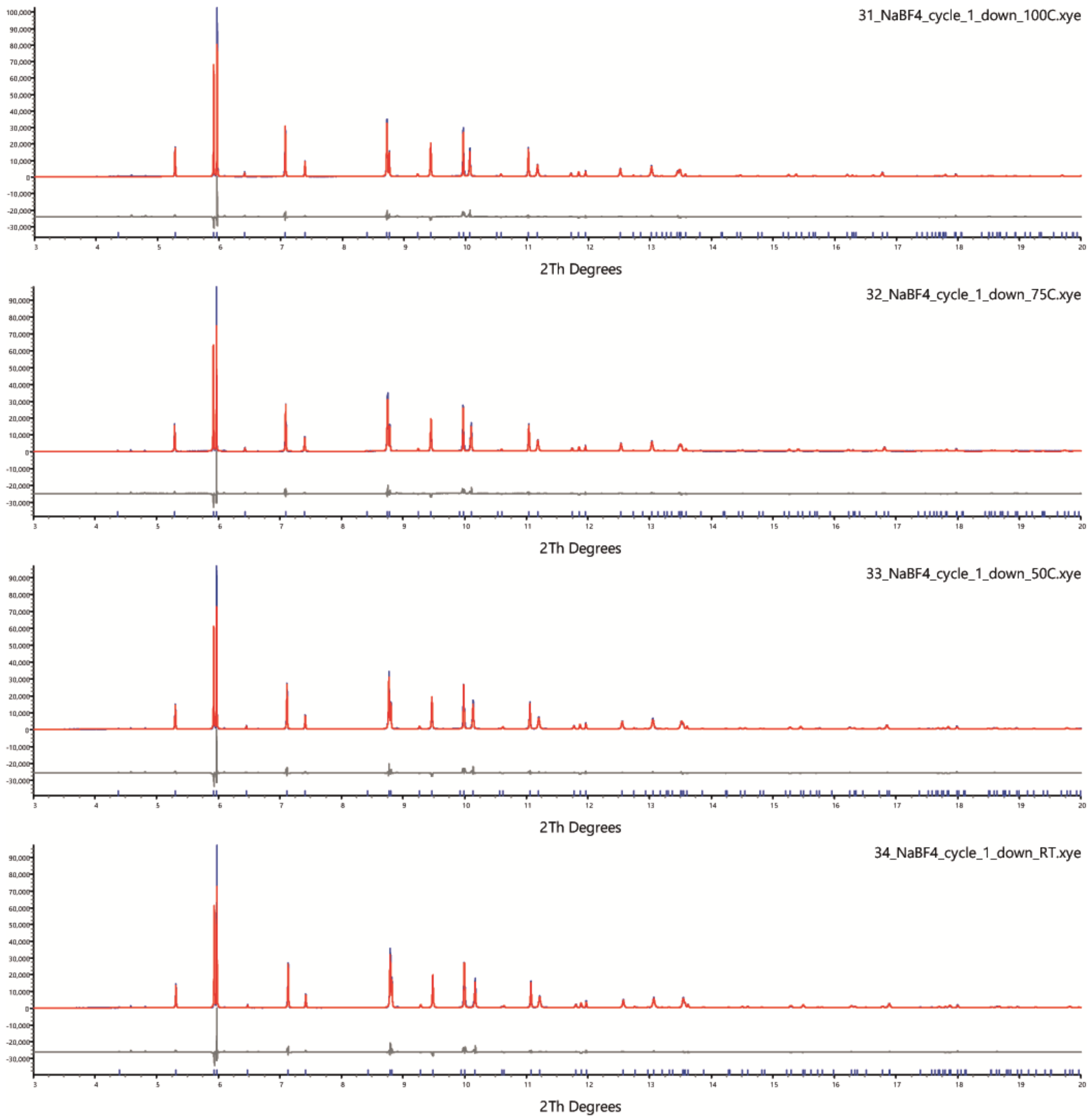

Figure S3 Comparison of the lattice parameters and unit cell volume of LT- and HT-KBF_4 from Cycle 1(squares) and Cycle 2 (circles) experiments. Values are obtained from Rietveld refinement of synchrotron powder diffraction data. For ease of comparison, the unit cell volume per formula unit has been shown.

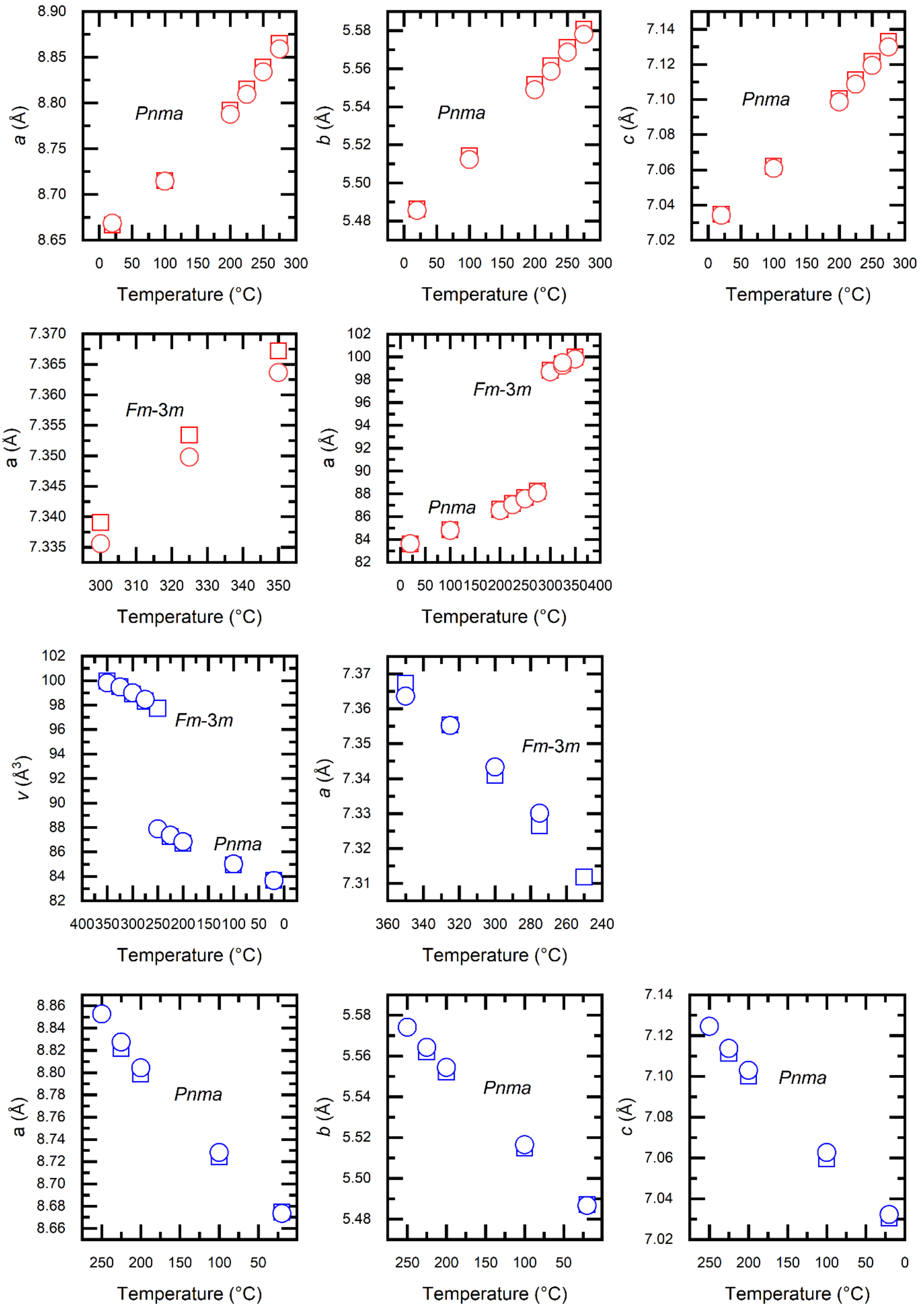

Figure S4 Comparison of the lattice parameters and unit cell volume of LT- and HT-$NaBF_4$ from Cycle 1(squares) and Cycle 2 (circles) experiments. Values are obtained from Rietveld refinement of synchrotron powder diffraction data. For ease of comparison, the unit cell volume per formula unit has been shown.

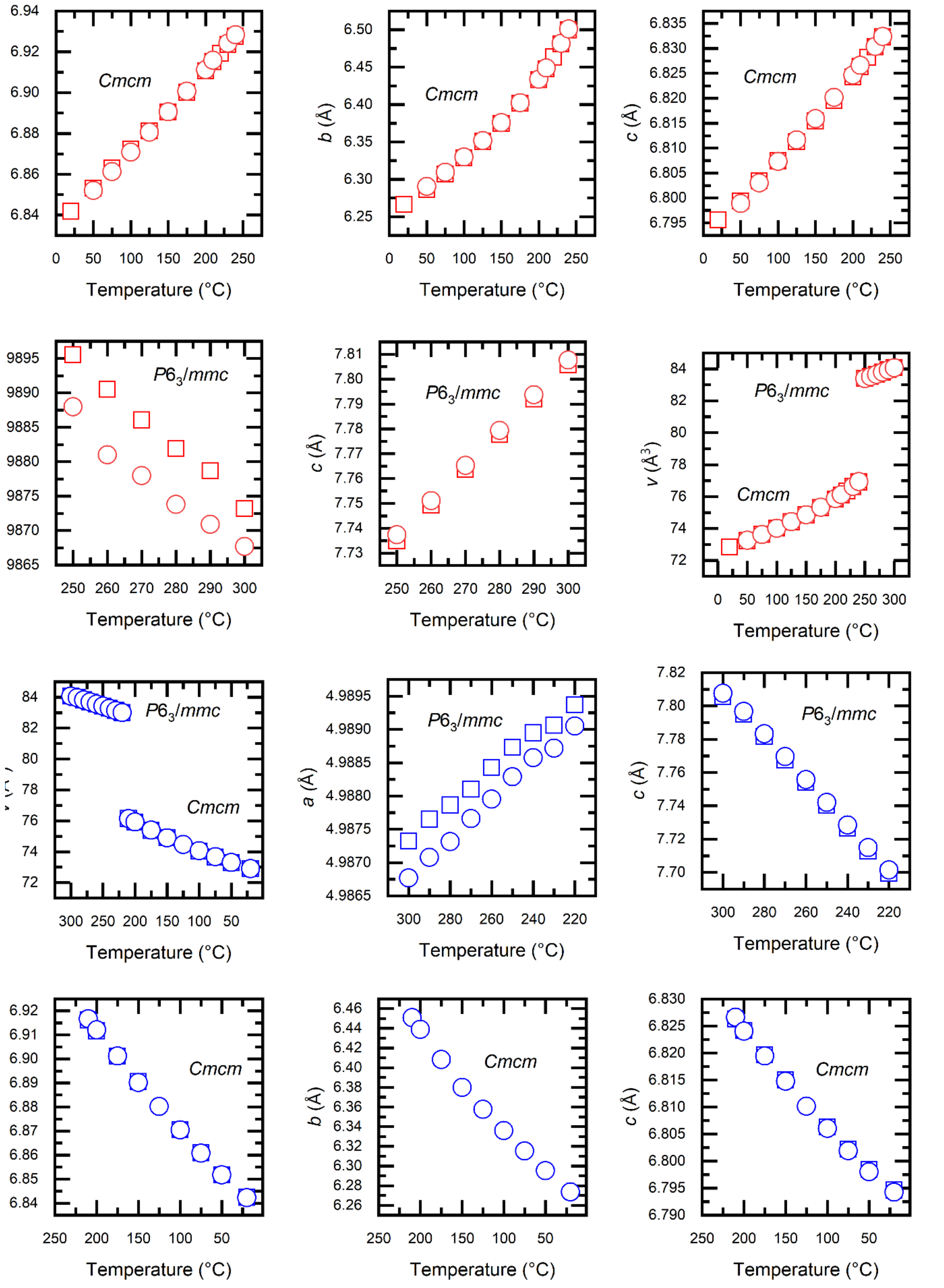

Figure S5 F atom real-space distribution throughout KBF_4-*Pnma* NVT-MD. K/B/F atom positions are projected from the MD supercell back on the conventional unit cell of the cubic phase (but note gamma angle has not been allowed to relax in NVT ensemble). Purple/green spheres show averaged positions of K/B atoms, with partial occupancy, isosurfaces show F atom density. Left/right panel shows the equilibrium distribution of F atoms throughout the MD at 300/600K. At both temperatures, F atoms preferentially take up specific positions around B atoms. At T=300 K each F atom remains on its site, while at T=600 K each BF_4 unit rotates. This re-orientational dynamics is not visible from these plots, but clear from the MSD.

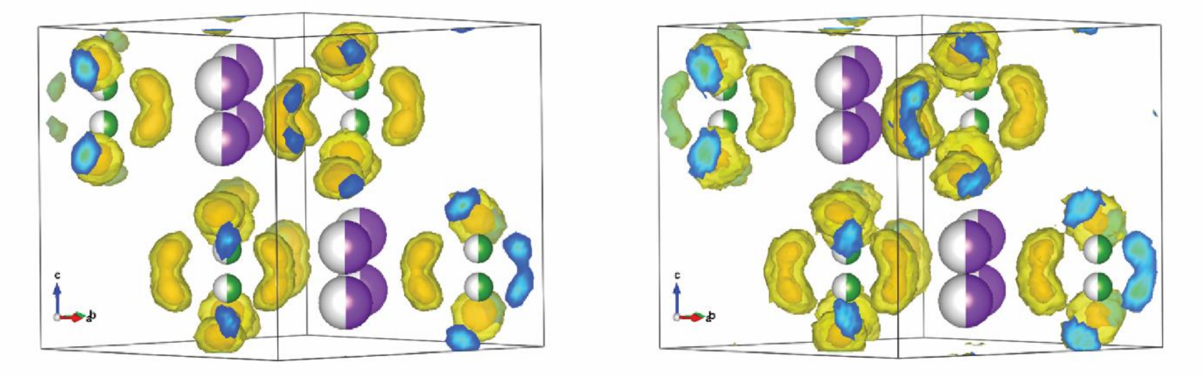

Figure S6 MSD(t) for F atoms relative to their bonded B atoms from HT-MD NPT runs at various temperatures as labelled; a) for $NaBF_4$-*Cmcm* phase, and b) for $NaBF_4$-*P*6_3/*mmc* phase.

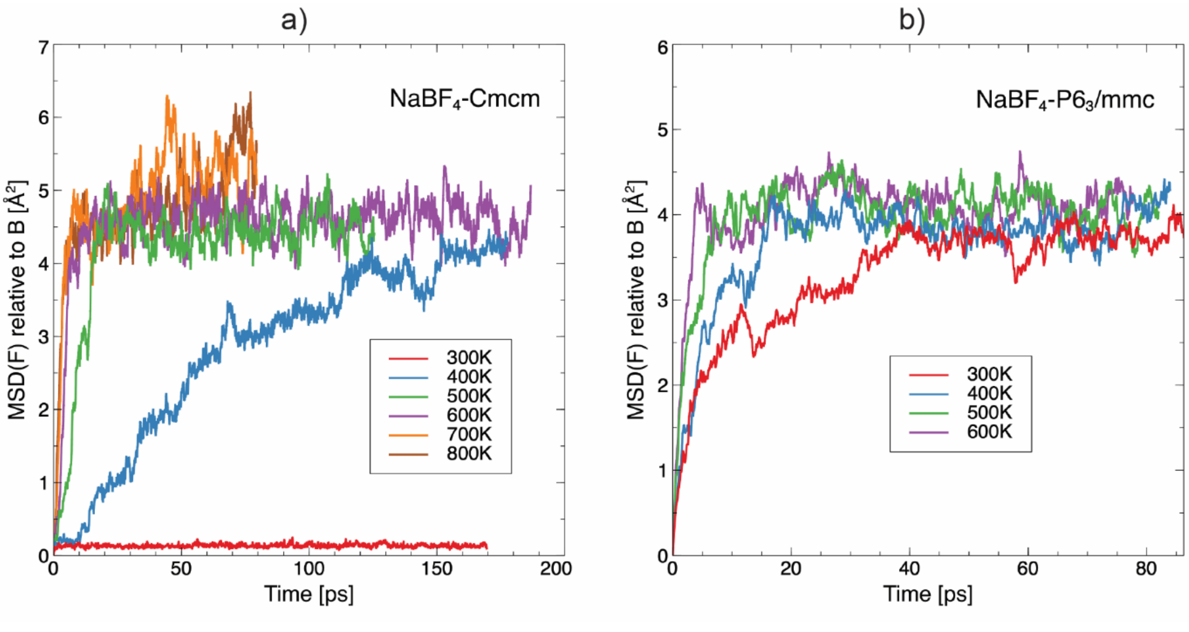

Figure S7 Averaged lattice lengths extracted from the HT-MD simulations and projected back onto the primitive $P6_3/mmc$ unit cell.

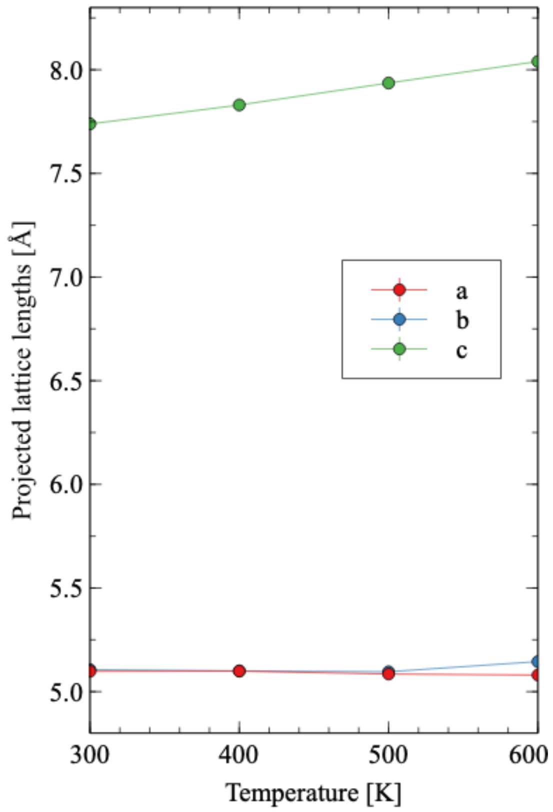

Figure S8 Partial distribution functions (PDF's) for NPT simulations of KBF_4 and $NaBF_4$ at a set of temperatures as indicated in the different panels. Note the strongly localised peak for the B-F covalent bond, which integrates to four F atoms around each B atom at all temperatures. For KBF_4, the orientational dynamics is visible in loss of medium range order in the PDF(B-F) for non-bonded interactions (r≥3Å), where the strong dip at 5.2Å disappears between 400 and 500K. For $NaBF_4$, the medium range order loss is not clear.

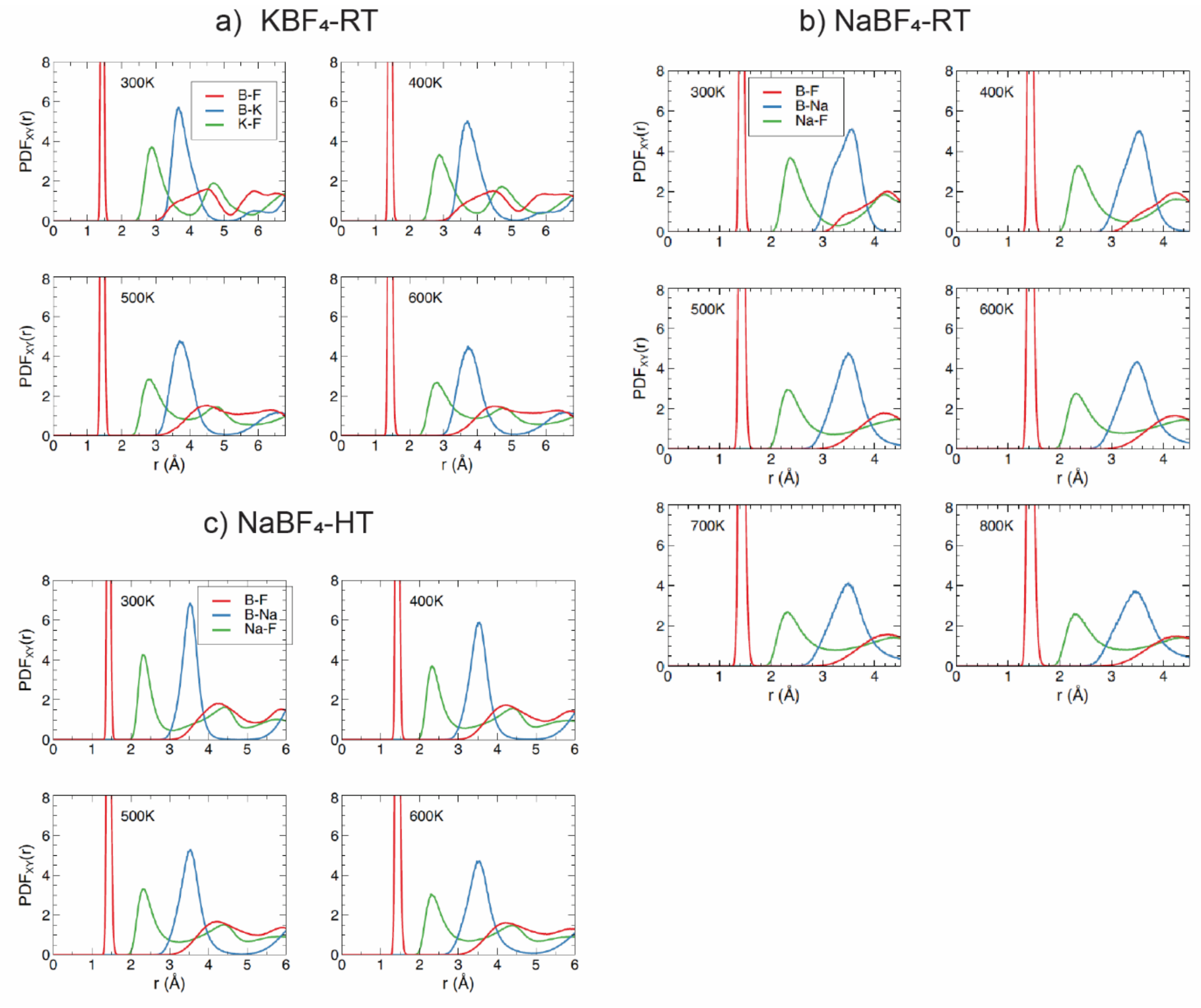

Supplement: Supplementary file 1 — cm3c02039_si_001.pdf [file cm3c02039_si_001.pdf]
